# Interaction of the gas vesicle proteins GvpA, GvpC, GvpN, and GvpO of *Halobacterium salinarum*

**DOI:** 10.3389/fmicb.2022.971917

**Published:** 2022-07-29

**Authors:** Alisa Jost, Felicitas Pfeifer

**Affiliations:** Microbiology and Archaea, Department of Biology, Technical University Darmstadt, Darmstadt, Germany

**Keywords:** *Haloferax volcanii*, split-GFP analysis, pulldown assays, cellulose-binding domain, gas vesicle proteins

## Abstract

The interactions of the four gas vesicle proteins GvpA, C, N, and O were investigated by split-GFP and pulldown assays. GvpA forms the ribs of the gas vesicle shell, whereas GvpC is attached to the exterior surface and stabilizes the gas vesicle structure. The AAA-ATPase GvpN as well as GvpO is found in much lower amounts. GvpN and GvpO formed homodimers and also the GvpN/GvpO heterodimer; both interacted with the C-terminal domain of GvpC when tested by split-GFP. When analyzed by pulldown assays, GvpN and GvpO also selected GvpA. The N-and C-terminal fragments of GvpC dimerized as Cterm/Cterm and Cterm/Nterm, but not as Nterm/Nterm. These interactions at both termini might lead to a network of GvpC molecules at the gas vesicle surface. However, a GvpA/GvpC interaction was not detectable, suggesting that the contact of both proteins is either mediated by another Gvp, or requires different structures that might form when GvpA is aggregated in the gas vesicle shell. Interactions of GvpA, C, N, and O were also studied with the accessory proteins GvpF through GvpM by split-GFP. GvpN bound GvpL only, whereas GvpO interacted with GvpF, I, and L, and the C-terminal domain of GvpC contacted GvpF, H, I, and L. GvpA/GvpA interactions were difficult to detect by split-GFP, but GvpA selected except for GvpI, K, and L all other accessory Gvp in pulldown assays. We will discuss the implications of these findings on gas-vesicle assembly.

## Introduction

Gas vesicles are intracellular hollow protein structures produced by several bacteria and archaea. The shell of the gas vesicles is devoid of lipids and formed entirely from proteins. These gas-filled nanobodies exhibit a spindle or a cylindrical structure closed at the ends by conical caps. Among bacteria, cyanobacteria such as *Anabaena, Planktothrix, Calothrix,* or *Microcystis*, the proteobacterium *Serratia* ATCC 39006, or the sporulating *Bacillus megaterium,* and *Streptomyces* produce gas vesicles (Walsby, 1994; Li and Cannon, 1998; Mlouka et al., 2004; van Keulen et al., 2005; Tashiro et al., 2016). In case of archaea, gas vesicle formation is confined to Euryarchaeota such as *Methanosarcina barkeri, Halobacterium salinarum, Haloquadratum walsbyi,* and *Haloferax mediterranei* (Pfeifer, 2012). Gas vesicles allow microbes to float in their aqueous environment and stay at positions more favorable for growth. The photosynthetic cyanobacteria and *Hbt. salinarum* are able to convert light into chemical energy, and light intensity, oxygen tension, temperature as well as nutrition influence gas-vesicle production. High light intensities and low temperatures of 15°C lead to a higher production of gas vesicles in the Haloarchaea (Bleiholder et al., 2012), whereas low light intensities of 200 lux and 38°C result in a 6- to 11-fold increased transcription of the gas vesicle protein (*gvp*) gene cluster of *Anabaena* sp. PCC 7120 (Cai et al., 2020). The expression of the *gvp* genes is usually regulated by endogenous transcriptional activators or repressing proteins (Ramsay et al., 2011; Pfeifer, 2012). In Haloarchaea, these are the GvpD and GvpE proteins, but also different basal transcription factors as well as the 5′-untranslated regions of the mRNAs influence the *gvp* gene expression during growth (Hofacker et al., 2004; Teufel et al., 2008; Bleiholder et al., 2012; Born and Pfeifer, 2019).

The haloarchaeal *gvp* gene cluster encoding the Gvp proteins involved in gas vesicle formation consists of 14 genes arranged in two or three clusters the rightward *gvpACNO* plus the leftward *gvpDEFGHIJKLM* (Englert et al., 1992a; DasSarma, 1993). The latter gene cluster could be also divided into *gvpDE* and *gvpFGHIJKLM* transcription units due to an additional promoter *P_pF_* upstream of *gvpF*. The *gvpA* and *gvpD* genes are oppositely oriented, and the BRE elements of the *P_pA_* and *P_pD_* promoters are separated by 35 bp only (Marschaus and Pfeifer, 2012). Eight of the Gvp proteins are essential, namely GvpA, F, G, J, K, L, M, and O (DasSarma et al., 1994; Offner et al., 2000). A deletion of one of the non-essential genes either alters the gas vesicle shape (*∆gvpC, ∆gvpI,* or *∆gvpN*), the gas vesicle strength (∆*gvpC* or ∆*gvpH*), or the amount of gas vesicles formed (*∆gvpN or ∆gvpDE*). The identification of essential genes has been done by single *gvp* gene deletions in transformants of the gas-vesicle negative species *Hfx. volcanii* (Offner et al., 2000). The expression of the *gvp* gene cluster leads to the spindle-shaped gas vesicles in *Hbt. salinarum* PHH1 (p-vac region) and starts with the transcription of *gvpFGHIJKLM* and *gvpACNO* in early exponential growth; GvpA and GvpC are gas vesicle structural proteins produced throughout growth, whereas the accessory proteins GvpF through GvpM are produced in minor amounts in early exponential growth only (Offner and Pfeifer, 1995). Protein–protein interaction studies by split-GFP done so far determined GvpF as the sole interaction partner of GvpA (Völkner et al., 2020). A crystal structure of the *Microcystis* GvpF protein has been obtained, and the protein is located at the gas-facing surface of the gas vesicle shell (Yang et al., 2015). Homologs of the haloarchaeal GvpF, J, K, L, and N are present in the cyanobacterium *Anabaena flos-aquae* (Kinsman and Hayes, 1997).

The hydrophobic GvpA (7–8 kDa) is the major constituent of the gas vesicle shell, and the sequence is highly conserved in archaea and bacteria. GvpA forms a monolayer by aggregating into 4.6 nm wide ribs running as low-pitch helix perpendicular to the long axis (Walsby, 1994; Offner et al., 1998). The shell is rigid and permeable to gases (Walsby, 1969). Since the gas-facing surface is hydrophobic, the precipitation of liquid water is excluded. *In silico* structural modeling of GvpA suggests a coil-α-β-β-α-coil structure (Strunk et al., 2011; Ezzeldin et al., 2012), and solid-state NMR studies imply that the aggregation within a rib occurs *via* the two antiparallel hydrophobic β-sheets (Sivertsen et al., 2010; Daviso et al., 2013). The amino acid sequence of the hydrophilic GvpC is much less conserved between bacteria and archaea; the sequence contains several 33–40 aa long repeats with predicted α-helical structure (4–5 in cyanobacteria, and 6–7 less conserved repeats in Haloarchaea; Walsby, 1994). The entire GvpC might form a long α-helical rod (Dunton et al., 2006). GvpC is found at the exterior surface of the gas vesicle shell and strengthens the structure (Hayes et al., 1988, 1992; Walsby and Hayes, 1988; Englert and Pfeifer, 1993; Halladay et al., 1993; Kinsman et al., 1995). The critical pressure required to collapse gas vesicles is 3-fold decreased when GvpC is washed off, and the addition of GvpC restores the original strength. GvpC is not essential since *Hfx. volcanii* ∆C transformants, expressing except for *gvpC* all other *gvp* genes of the p-vac region, contain large, but deformed gas vesicles (Offner et al., 1996). The diameter and critical collapse pressure of cyanobacterial gas vesicles are correlated with GvpC proteins of different numbers of repeats (Dunton and Walsby, 2005); a higher number of the repeats in GvpC results in larger and less stable gas vesicles.

Recently, gas vesicles became an attractive object for biomedical studies, and GvpC is an important target to modify their properties. By fusion of foreign peptides or proteins to the C-terminus of GvpC, the gas vesicles can be decorated at the surface with additional proteins (DasSarma and DasSarma, 2015). Proteins of viruses or pathogenic bacteria have been fused to the C-terminus of GvpC of *H. salinarum*, and the modified gas vesicles, not toxic to animals or humans, were injected into animals for antibody production (Stuart et al., 2001; DasSarma et al., 2015). Modified and functionalized gas vesicles are also used as stable contrast agents for ultrasound and magnetic resonance imaging (MRI) in biomedical research; they enable harmonic, multiplexed, and multimodal ultrasound imaging (Shapiro et al., 2014; Lakshmanan et al., 2017). Alterations of GvpC by fusion of foreign peptides lead to a modulation of the acoustic properties enabling multimodal imaging or targeting to visualize certain cell types (Lakshmanan et al., 2016). Imaging becomes very sensitive when gas vesicles are filled with ^129^Xe, and their location in living mice is detectable in picomolar concentrations (Farhadi et al., 2018). Gas vesicles also enable an efficient neuromodulation by low-intensity ultrasound. The gas vesicles of *Anabaena flos-aquae* have been used as localized force actuators in a targeted mouse brain region, and improved the precision of low-intensity ultrasound stimuli of proximate neurons (Hou et al., 2021). Therefore, studies on the gas vesicle structures and the principles of assembly are of high interest.

**Figure 1:**
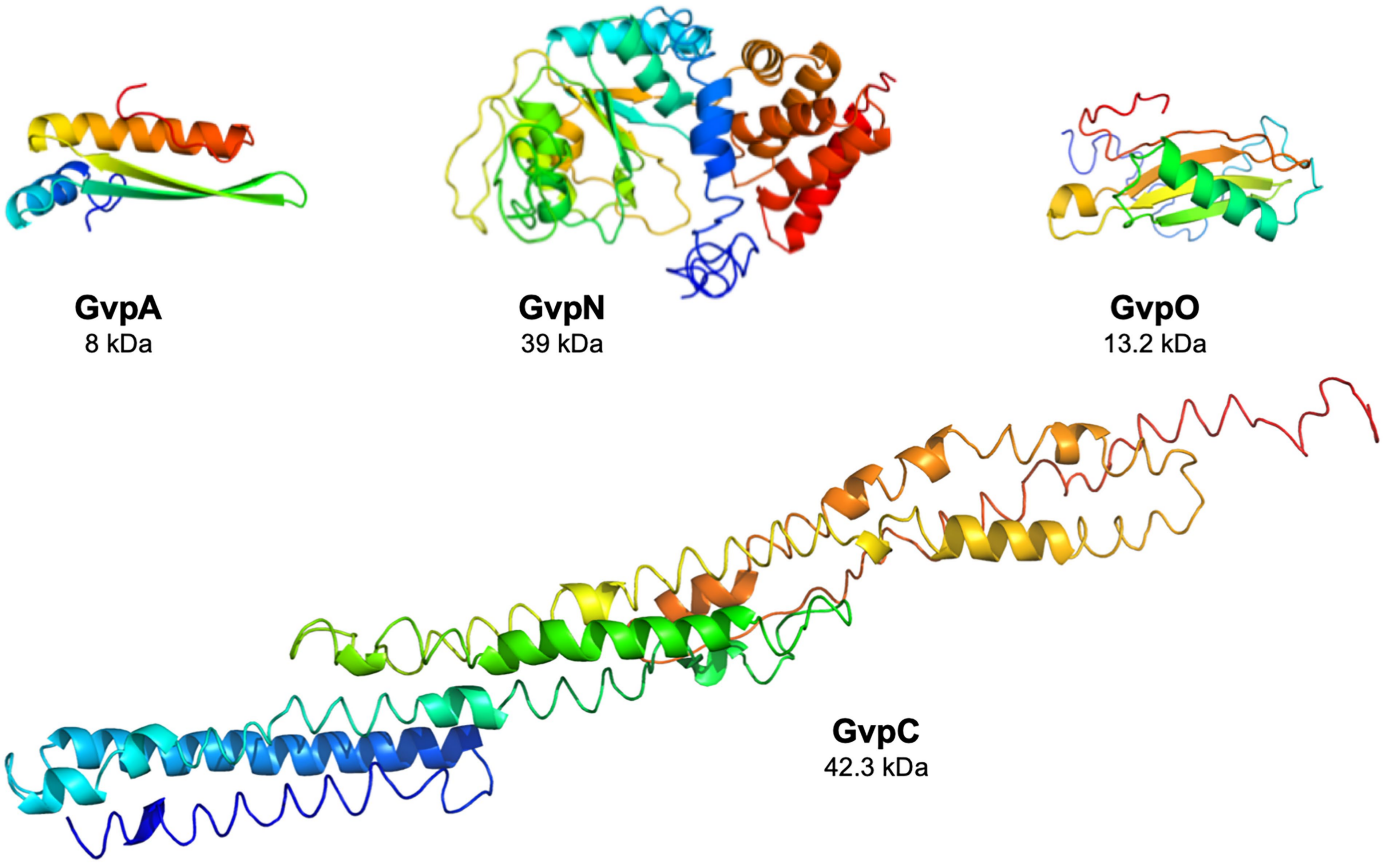
Structural models of the four Gvp proteins. The structural model of GvpA has been obtained *in silico* (Strunk et al., 2011), and the structural models of the other proteins were calculated by the I-Tasser server (Zhang, 2008; Roy et al., 2010; Yang et al., 2015).

The functions of the eight accessory Gvp proteins encoded by *gvpFGHIJKLM* during gas-vesicle assembly are not known so far. However, all of these proteins interact and might form (a) nucleation complex(es) at the initiation and/or early steps of the assembly since their co-transcript is produced early in growth (Offner and Pfeifer, 1995; Völkner et al., 2020; Jost et al., 2021). The 32-kDa GvpL binds any of these accessory Gvp and might function as platform to bring them altogether. However, the interactions of the GvpA, C, N, and O were not investigated so far. The *gvpACNO* operon encodes the two major gas vesicle structural proteins, GvpA and GvpC, as well as the AAA-ATPase GvpN. The function of the 13.2 kDa GvpO is not yet known, but the protein is essential since ∆O transformants lack gas vesicles (Vac^−^; Offner et al., 2000). In the absence of GvpN, only tiny gas vesicles are formed although GvpA and GvpC are produced in normal amounts (DasSarma et al., 1994; Offner et al., 2000). GvpN contains a Walker motif and hydrolyzes ATP as shown for *Anabaena* sp. PCC 7120 (Cai et al., 2020). It is likely that the ATPase activity is required during gas-vesicle assembly. GvpN is produced in much lower amounts than GvpC or GvpA, whereas the Haloarchaea-specific *gvpO* is expressed as leaderless transcript from the promotor *P_pO_* throughout growth (Born and Pfeifer, 2019). Structural models obtained by I-TASSER server for the four proteins are shown in Figure 1.

Here, we investigated the interactions of GvpA, C, N, and O *in vivo* by split-GFP analyses as well as pulldown assays using the cellulose-binding domain of *Clostridium thermocellum*, CBD, as tag. *In vitro* interaction studies with tagged Gvp proteins synthesized in *Escherichia coli* often lead to the problem that the salt-adapted proteins denature and precipitate in the low-salt environment. Synthesis in Haloarchaea circumvents this problem, but the amounts of the proteins produced are much lower than in *E. coli,* since strongly inducible promoters are not available. The *in vivo* interaction studies performed in this report in *Hfx. volcanii* demonstrated interactions for all four proteins. GvpN and GvpO formed homodimers, a heterodimer, and also bound to the C-terminal domain of GvpC (Cterm). The dimer formation of GvpC was tested with the N-terminal and C-terminal fragments Nterm and Cterm by split-GFP, and interactions were found. However, the GvpC/GvpA interaction was not detectable. The four Gvp proteins were also analyzed for interactions with GvpF through GvpM.

## Materials and methods

### Strains and cultivation conditions

*Escherichia coli* strains Top10F (Invitrogen, Life Technologies) and GM1674 (*dam^−^*) (Palmer and Marinus, 1994) were grown in Luria-Bertani (LB) medium supplemented with 100 μg/ml ampicillin at 37°C overnight. Growth on solid media was performed overnight on LB media supplemented with 1.5% agar. *Haloferax volcanii* WR340 (*his* mutation; Bitan-Banin et al., 2003) was incubated in 3 M VM medium (3 M NaCl, 150 mM MgSO_4_, 50 mM KCl, 0.05% (w/v) CaCl_2_, 25 mM Tris–HCl pH 7.2, 10 nM MnCl_2_, 0.5% (w/v) tryptone, 0.3% (w/v) yeast extract) supplemented with 0.02% (w/v) histidine. Transformation was done as described (Pfeifer and Ghahraman, 1993), and transformants were selected on solid media supplemented with 6 μg/ml mevinolin (selection of plasmid pWL102 or pWL_fdx_) and/or 0.2% novobiocin (selection of pJAS35). For solid media, 1.8% agar was added; the plates were incubated at 42°C for 5–7 days in a plastic bag including a wet paper to maintain a humid atmosphere and prevent the formation of salt crystals. Colonies examined for the Vac phenotype were grown for 5 weeks at room temperature in the dark. Liquid cultures were grown at 42°C, 180 rpm for 3–4 days. Cultures used to quantify the fluorescence by split-GFP were incubated for 24 h at 37°C and 180 rpm, followed by 24 h at 30°C (Winter et al., 2018). *Hfx. volcanii* cultures used for pulldown experiments were grown to OD 1.5–2.0 at 37°C and 180 rpm.

### Vector constructions

For pulldown experiments, the vectors _CBD_A, _CBD_C, _CBD_N, and _CBD_O were constructed containing the *cbd* reading frame encoding the cellulose-binding domain fused to the 5′-end of the respective *gvp*. In addition, *cbd* was fused at the 3′-end of *gvp* (A_CBD_, C_CBD_, N_CBD_, and O_CBD_). These fusions were inserted in pWL_fdx_ and expressed under *P_fdx_* promoter control. For the constructions, vector pCBD was used containing the *cbd* reading frame of plasmid pWL-CBD-sec11b (Fine et al., 2006) in pWL_fdx_. The *cbd* reading frame is surrounded by NcoI and BamHI restriction sites (5′ to *cbd*), and XbaI and KpnI sites (3′ to *cbd*) allowing the insertion of the respective *gvp* reading frame to yield the X_CBD_ or _CBD_X fusion proteins (Völkner et al., 2020). The different *gvp* sequences were amplified by PCR using the p-vac region inserted in pWL102 as a template (Offner et al., 2000). The oligonucleotides used for these experiments are listed in Supplementary Table S1.

For the split-GFP analysis, the previously described vectors pJAS-NGFP-Nterm (_N_X – NGFP fused at the N-terminus of GvpX) and Cterm (X_N_ – NGFP fused at the C-terminus), as well as pWL_fdx_-CGFP-Nterm (_C_X) and pWL_fdx_-CGFP-Cterm (X_C_) were used (Winter et al., 2018). These vectors either contain the N-terminal or C-terminal portion of the *mgfp2* reading frame encoding the salt-stable green-fluorescent protein mGFP2 (Born and Pfeifer, 2019). The split in the mGFP2 sequence is between amino acid (aa) 157 and 158, resulting in the N-terminal (NGFP) and C-terminal (CGFP) portions, respectively. The fusion of *mgfp2* fragments to *gvp* includes a 14 aa (pJAS) or 16 aa (pWL_fdx_) linker region (Winter et al., 2018). In both vectors, the reading frames are expressed under the control of the constitutive *P_fdx_* promoter. The vectors containing *gvpA*, or fragments A1-22, A1-34, A1-43, A20-47, and A44-76, have been described previously (Winter et al., 2018; Völkner et al., 2020). The vectors containing *gvpF_mut_, gvpC*, *gvpN*, and *gvpO*, or the GvpC fragments Cterm and Nterm, were produced in this report. The respective *gvp* sequences were amplified by PCR using the p-vac region as template. The oligonucleotides used are listed in Supplementary Table S1.

Construct _N_A + pX (X = C, N, O, NO, CNO) contains the *gvpA* reading frame inserted *via* NcoI and BlpI in vector pP2-NGFP-Nterm; the fusion is expressed under the control of *P_fdx_*. The *ngfp* reading frame is fused upstream of *gvpA*. The *gvpX* reading frame(s) is (are) inserted *via* HindIII and SpeI in the same vector molecule and expressed under *P2* promoter control. Construct A_N_ + pX (X = C, N, O, NO, CNO) contained *gvpA* inserted *via* NcoI and BlpI in vector pP2-NGFP-Cterm. The NGFP reading frame is located downstream of *gvpA* and thus fused to *gvpA* at the 3′-end. Similarly, the *gvpX* reading frame(s) is (are) inserted *via* HindIII and SpeI and expressed under *P2* promoter control in the same vector molecule. In all cases, the correct insertion was verified by DNA sequence analysis. The DNA was demethylated by passage through *E. coli* GM1674 (*dam^−^*) (Palmer and Marinus, 1994) to avoid a restriction barrier in *Hfx. volcanii*. *Haloferax volcanii* WR340 was transformed and the presence of the plasmids confirmed by PCR. The presence of the respective Gvp protein was determined by Western analysis using the respective antiserum.

Construct ∆F contains except for *gvpF* all genes of the p-vac region of *Hbt. salinarum* inserted in pWL102 (Lam and Doolittle, 1989). The vector construction was done using Gibson assembly (Gibson et al., 2009, 2011). The *gvpF* reading frame was deleted in such a way that the first and the last three amino acids of the reading frame are still present. Complementation of ∆F in *Hfx. volcanii* WR340 transformants was performed with *gvpF* (wild type) or *gvpF_mut_* (variant) inserted in vector pJAS35 (Pfeifer et al., 1994). The mutagenesis of the *gvpF* reading frame inserted in pBSKII+ was performed in *E. coli*. The substitutions of charged amino acid residues of GvpF are E03A/R, E12A/R, E14A/R, D15A/R, E17A/R, D19A/R, E21A/R, E27A/R, D45A/R, D46A/R, E50A/R, R51A/E, D53A/R, E54A/R, D55A/R, E57A/R, E65A/R, K68A/E, E70A/R, E71A/R, E72A/R, R73A/E, K85A/E, R88A/E, K91A/E, R95A/E, R98A/E, R102A/E, D123A/R, D124A/R, D154A/R, R155A/E, D184A/R, E185A/R, and R213A/E. The nucleotides encoding the desired substitution were introduced by site-directed mutagenesis PCR. The oligonucleotides containing the desired alteration are listed in Supplementary Table S1. In all cases, the desired mutation was confirmed by DNA sequence analysis.

### Western analysis

The presence of the Gvp proteins was determined by Western analysis. The cultures were grown in 50 ml of 3 M VM (+His) media at 42°C. The cells were harvested in the early stationary growth phase (centrifugation at 2,370 × *g*, 30 min, 4°C), resuspended in lysis buffer plus 0.1 mg/ml DNase I, and incubated for 3 h at 37°C, followed by dialysis against Tris–HCl, pH 7.2 overnight to remove the salt. The cell debris was removed by centrifugation, and the protein concentration in the supernatant was determined by Bradford assay. Twenty microgram of protein was separated by SDS-PAGE (Schägger and von Jagow, 1987) and transferred on a PVDF membrane (Roti Fluoro PVDF, Carl Roth). The dried membrane was reactivated with 100% (v/v) methanol and washed with PBS (137 mM NaCl, 2.7 mM KCl, 10 mM Na_2_HPO_4_, and 2 mM KH_2_PO_4_) before blocking with Odyssey Blocking Buffer (Licor) for 1–2 h. The membrane was incubated overnight with the respective antiserum, washed four times for 5 min with PBS + 0.1% Tween20 (v/v), and incubated for 2–3 h with the secondary antibody IRDye 800 CW (Licor) coupled to a fluorophore. After washing the membrane 4 times for 5 min with PBS + 0.1% Tween20, excess Tween20 was removed by washing with PBS. Detection of the secondary antibody was performed at 800 nm using Odyssey Fc Imager (Licor). The blots are always inverted to black-white.

The Gvp antisera have been already described (Englert et al., 1992b; Englert and Pfeifer, 1993; Offner and Pfeifer, 1995; Tavlaridou et al., 2013, 2014; Völkner et al., 2020;). For this, recombinant his-tagged Gvp proteins have been isolated from *Escherichia coli* lysates by Ni-NTA affinity chromatography and used for the generation of antisera in rabbits (Seqlab, Göttingen, or Eurogentec, Köln). In the case of GvpF and GvpI, synthetic peptides were used (Dissertation Völkner, 2020; Eurogentec Köln).

### Pulldown assays using CBD-tagged proteins

Each one of the four Gvp proteins was fused to the cellulose-binding domain, CBD, at the N- or C-terminus (_CBD_X or X_CBD_) and lysates of the respective _CBD_X/Y transformants were tested in pulldown assays (X, Y = A, C, N, or O). In the case of _CBD_A, also _CBD_A/F-M transformants were tested. The pulldown assays were done as described by Völkner et al. (2020). In each case, 400 ml of cultures was grown at 37°C, 180 rpm to the late exponential growth phase. The harvested cells were resuspended in 5 ml lysis buffer (2.5 M KCl, 50 mM MgCl_2_, 1 mM EDTA, 5% (v/v) glycerol, 50 mM Tris–HCl pH 8.0) plus 0.1 mg/ml DNase I. The cells were lysed by ultrasound (Branson Sonifier 250) and the suspension was cleared for 20 min at 2,370 × *g*, 4°C. The soluble protein fraction (7 ml) was incubated with 1 ml of a 10% (w/v) cellulose suspension (Avicel PH-101, Sigma Aldrich) for 30 min at room temperature on an overhead rotator. The cellulose matrix plus bound proteins were recovered by centrifugation (2,370 × *g*, 30 s) and resuspended in 600 μl washing buffer (2.5 M KCl, 50 mM Tris–HCl pH 8.0). The solution was transferred to a Mobicol column (Mobitec) and washed six times with 600 μl washing buffer. Gvp proteins were eluted by resuspension in 500 μl 100% ethylene glycol, 1 min at room temperature, and centrifugation at 4,700 × *g*, 5 min. All fractions were dialyzed against 10 mM Tris–HCl, pH 7.2 overnight, and 15 μl of each fraction was used for SDS-polyacrylamide gel electrophoresis, SDS-PAGE (Schägger and von Jagow, 1987). Gvp proteins were detected by Western analysis using the respective antisera.

### Quantitation of GFP fluorescence

*Haloferax volcanii* cultures of the respective X/Y transformants, producing the two Gvp proteins fused to NGFP or CGFP under investigation, were grown to OD_600_ 1.5–2 (48 h) in a two-step incubation (24 h at 37°C followed by 24 h at 30°C; Winter et al., 2018). Two milliliter of the culture was harvested for 2 min at 9,600 × *g*, 20°C, the sediment was washed with 1 ml of basal salts (3 M NaCl, 150 mM MgSO_4_, 50 mM KCl), and the cells were resuspended in 500 μl of basal salts. The cell concentration was adjusted to OD_600_ 1, and 300 μl was transferred to a microtiter plate. The fluorescence was measured with a phosphor imager in LAU/mm^2^. The relative fluorescence was calculated as described (Winter et al., 2018). The original data of the experiments described in this report are presented in Supplementary Table S3.

### Isolation of gas vesicles and transmission electron microscopy

Gas vesicles were isolated from colonies grown on solid media for 5 weeks. A few of the colonies were transferred to 1 ml of 1 mM MgSO_4_ containing 10 μg/ml DNase I to lyse the cells and release the gas vesicles (3 h on an overhead rotator). The mixture was centrifuged for 2 h at 95 × *g* and 4°C. Gas vesicles float to the surface and settle as a white layer. They were removed with a pipette and transferred to 500 ml of 100 mM Tris–HCl, pH 7.2 plus 5% NaCl for transmission electron microscopy.

Cells of the ∆F + F_mut_ transformants or isolated gas vesicles were inspected by TEM. Twenty microliter of gas vesicle suspension, or cells resuspended in 20 μl of basal salts, was transferred to a formvar-coated copper grid (300 mesh, Plano GmbH). The suspension was left on the grid for 1 min, and the liquid was removed with a Whatman 3 M paper. Images were taken using a Zeiss EM109 microscope and Gatan Multiscan 600 W camera.

## Results

The interactions of the four proteins GvpA, C, N, and O were investigated by two different methods, i.e., pulldown assays using proteins tagged with the cellulose-binding domain, CBD, and split-GFP analysis. For the analysis by split-GFP, one of the two fragments of the salt-stable green-fluorescent mGFP2, NGFP and CGFP, was fused to the N- or C-terminus of a Gvp protein under investigation. NGFP and CGFP reassemble to form a fluorescent mGFP2 only upon interaction of both Gvp proteins (Winter et al., 2018). The fluorescence can be quantified in the *Hfx. volcanii* transformants.

### Interactions of GvpA, GvpC, GvpN, and GvpO

**Figure 2:**
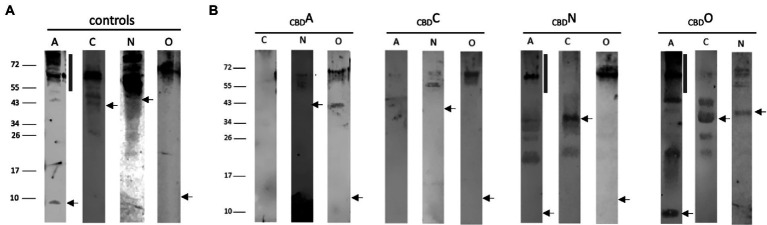
Western analyses of the pulldown assays using _CBD_A, _CBD_C, _CBD_N, or _CBD_O. In each case, 15 μl of the elution sample was separated by SDS-PAGE, the proteins were transferred to a PVDF membrane, incubated with the respective antiserum, and visualized by the fluorophore-labeled secondary antibody IRDye 800 CW (Licor). The blots are inverted to black-white. Numbers on the left indicate sizes in kDa. **(A)** Western analysis of *Hfx. volcanii* transformants producing the GvpA, C, N, or O used as control. The apparent masses of the protein monomers are 8.0 kDa, GvpA; 42.3 kDa, GvpC; 39.0 kDa, GvpN, and 13.2 kDa, GvpO. The position of the respective monomer is indicated by an arrow. The vertical bar in the panel of GvpA marks the position of multimers formed by this hydrophobic protein. **(B)** Western analysis of *Hfx. volcanii*_CBD_X/Y transformants (X, Y = A, C, N, or O) containing the CBD-tagged bait protein plus one of the other three proteins as prey is indicated by the letter on top of each blot. Only the elution fraction of the cellulose matrix is shown. In each case, the arrow marks the position of the respective Gvp monomer, and the vertical bar the position of GvpA multimers.

For pulldown assays, the 21-kDa cellulose-binding domain CBD (Fine et al., 2006) was fused to the N-terminus of GvpA, C, N, or O, resulting in the _CBD_A, _CBD_C, _CBD_N or _CBD_O fusion proteins that were used as bait to test the interactions in _CBD_X/Y transformants (X, Y = A, C, N, O). The respective transformants contained the *cbd-gvpA, cbd-gvpC, cbd-gvpN*, or *cbd-gvpO* fusions in vector pCBD (Völkner et al., 2020), and in addition, the expression vector pJAS35 that expresses the reading frame of the putative interaction partner. The _CBD_X/Y transformants were grown to an optical density of OD_600_ 2, the cells collected and lysed, and a cellulose matrix was used to pulldown the _CBD_X protein plus putative binding partner Y. After washing the matrix several times, the proteins bound were eluted, separated by SDS-PAGE and analyzed by Western analysis. Lysates of transformants producing GvpA, C, N, or O were used as controls (Figure 2A). The protein bands observed in the control samples were larger in size than calculated for the respective Gvp monomer and might constitute oligomers of these Gvp. Multimers were especially observed with the hydrophobic GvpA, with GvpN and the 13.2-kDa GvpO.

In the case of _CBD_A tested with GvpC, N, or O, the Western analyses of the elution samples of the _CBD_A/N or _CBD_A/O transformants yielded protein bands of 55–60 kDa (GvpN) and >72 kDa (GvpO), similar to the occurrence of these proteins in the control samples (Figure 2B). The results implied that both GvpN and GvpO were selected by _CBD_A. A smaller protein band (40 kDa) was observed with GvpO and might be due to the _CBD_A/O complex, whereas the larger band could also represent GvpO multimers. GvpC was not detectable in the elution sample of the _CBD_A/C transformant. Similarly, GvpA was not observed in the sample of _CBD_C/A transformants, suggesting that GvpC and GvpA are not able to interact (Figure 2B). In contrast, GvpN and GvpO were detectable in the _CBD_C/N and _CBD_C/O transformants and thus selected by _CBD_C (Figure 2B). The sizes of these bands were similar to the bands observed in the controls (Figure 2A). All three proteins were able to bind _CBD_N (Figure 2B). In the case of the _CBD_N/A transformant, the strongest GvpA protein band was observed at 60 kDa, and also smaller bands occurred, but the 8-kDa GvpA monomer was not detectable. The 60-kDa band, also present in the control sample, was most likely due to aggregated GvpA. Multimers of GvpA with sizes >150 kDa were only faintly detectable in the _CBD_N/A transformant (Figure 2B). In the case of the _CBD_N/C transformant, the 42-kDa GvpC monomer was found, suggesting that GvpN suppresses the formation of the GvpC dimer present in the GvpC control (Figure 2A). In case of the _CBD_N/O transformant, GvpO was detectable as large multimer >60 kDa – similar to all other samples containing GvpO (Figure 2B). In the case of _CBD_O, the _CBD_O/A elution sample contained the GvpA monomer as well as GvpA multimers up to the top of the separating gel (>150 kDa), suggesting that GvpO is able to bind GvpA as monomer as well as multimer. The pattern of GvpA differs from the pattern observed in the control sample that contained mostly large aggregates (Figure 2A). It is possible that GvpO stabilizes the GvpA monomer like a chaperon, but also binds GvpA multimers. In addition, _CBD_O selected GvpC and GvpN; in both cases, the monomers were observed (Figure 2B). An overview of these results is found in Table 1. Overall, these analyses showed that GvpA and GvpC interacted with GvpN and GvpO, but the GvpA-GvpC (A/C) interaction was not detectable.

**Table 1:** Interactions of Gvp proteins.

Gvp	Pulldown^*^ assay	Monomer/dimer/multimer formation^**^	Split-GFP^#^ analysis	Dimer/heterodimer, special observations
A	N, O	N/N, O–O	–	A/A only + CNO
C	N, O	N/N, O–O	**C**, N, O	Cterm: N, O
N	A, C, O	A/A, A–A, C_mono_, O–O	**N, O**, C	N/N highest rf
O	A, C, N	A_mono_, A–A, C_mono_, N_mono_, N/N	**N, O**, C	N/O highest rf

* Dimerization not tested in pulldown assays.

# Bold: rf > 10; grey: weak interaction.

** X/X, dimer; X–X, multimer; X_mono_, monomer.

**Figure 3:**
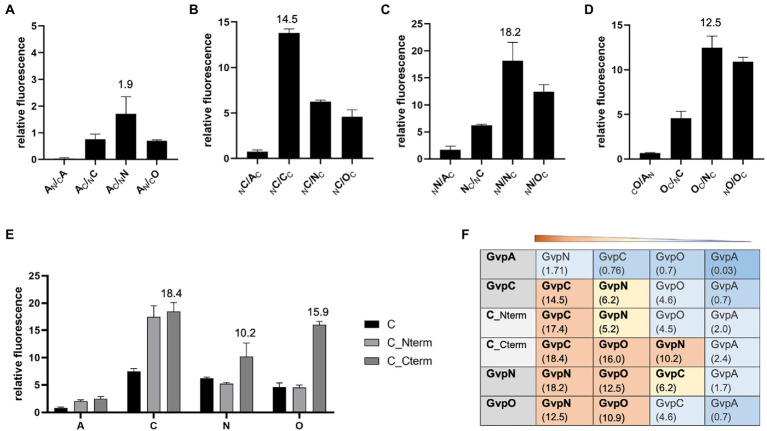
Split-GFP analyses to investigate the interaction of GvpA, C, N, and O. The relative fluorescence (rf) is given in LAU/mm^2^. In each case, only the combination resulting in the highest rf value is presented, and the position of the GFP-tag can be deduced from the place of the subscript N (for NGFP) or C (CGFP) at the left (N-terminal) or right side (C-terminal) of the respective Gvp protein. All experiments were performed in two biological and three technical replicates (*n* = 6). The relative fluorescence was calculated in relation to the fluorescence of *Hfx. volcanii* wild type. The error bars represent the standard deviation of the rf values of the biological/technical replicates. **(A)** Interaction of GvpA with GvpA, C, N, or O; **(B)** Interaction of GvpC with GvpA, C, N, or O; **(C)** Interaction of GvpN with GvpA, C, N, or O; **(D)** Interaction of GvpO with GvpA, C, N, or O; **(E)** Interaction of GvpC fragments Cterm and Nterm with GvpA, C, N, or O. **(F)** Overview of the rf values for each of these interactions. The Gvp proteins interacting with the respective Gvp protein placed on the left (shaded in grey) are ordered by rf values and shaded in red, rf > 10; yellow, rf < 10 to >5; blue, rf < 5; dark blue, rf < 1.

The second method applied to analyze protein–protein interactions was split-GFP, also used to investigate the formation of homodimers. The N-terminal fragment of mGFP2, NGFP, was fused to one of these Gvp at the N- or C-terminus, and the C-terminal fragment CGFP to the N- or C-terminus of the second Gvp protein. Upon interaction of both Gvp proteins in the transformants, the GFP fragments assemble a fluorescent mGFP2 that can be measured in a phosphor imager. Eight possible combinations of these fusion proteins were studied per interaction (_N_X/_C_Y, _N_X/Y_C_, X_N_/_C_Y, X_N_/Y_C_, _C_X/_N_Y, _C_X/Y_N_, X_C_/_N_Y, and X_C_/Y_N_, with X, Y = A, C, N, or O; the subscript C or N at the left or right indicates CGFP or NGFP fused at the N- or C-terminus). Only the result of the combination yielding the highest fluorescence of the respective transformants is presented in Figure 3. In the case of GvpA, the relative fluorescence obtained with the A/A, A/C, A/N, and A/O transformants was very low (rf < 2; Figure 3A). According to our previous analyses, a relative fluorescence of rf < 5 is regarded as a very weak interaction (Winter et al., 2018; Völkner et al., 2020). The A/A transformants (GvpA dimerization) yielded an almost undetectable fluorescence, and the highest relative fluorescence (rf 1.9) was observed for the A/N transformants. The highly hydrophobic GvpA has a strong tendency to form aggregates that presumably interfered with the split-GFP analysis depending on two soluble fusion proteins to assemble GFP upon interaction. In the case of GvpC, the C/C transformants yielded a high relative fluorescence (rf 14.5) indicative of GvpC dimers, but also C/N interactions were found and C/O was slightly below rf 5 (Figure 3B). A high fluorescence (rf > 10) was determined for the N/N or O/O transformants suggesting the formation of both dimers. Also, the fluorescence of the N/O transformants was high (rf 12.5), implying the formation of a heterodimer (Figures 3C,D). These results confirmed the data of the pulldown assays described above (Table 1).

The interaction of GvpC was investigated in further detail using two fragments encompassing the N-or C-terminal regions of GvpC. The GvpC protein of *Hbt. salinarum* contains seven related amino acid (aa) sequences (38–40 aa in length) starting at the N-terminus, and the globular C-terminal portion contains a zinc-finger motif. The N-terminal fragment Nterm (amino acids 10–130) harbors the first three α-helices, and the C-terminal fragment Cterm (aa 329–382) the globular domain. Both fragments interacted with GvpC (rf 17–18), demonstrating that GvpC is able to dimerize (or multimerize) *via* both regions (Figure 3E). Testing the two GvpC fragments for intramolecular interactions resulted in high rf values in the case of Cterm/Cterm (rf 20.7) and Cterm/Nterm (rf 10.1), whereas a much lower fluorescence was obtained for Nterm/Nterm transformants (rf 4.3). It appears that GvpC polymerizes *via* Cterm/Cterm and Cterm/Nterm interactions, whereas the Nterm/Nterm interaction is not preferred. Testing these fragments with GvpA yielded low fluorescence values (rf < 2.5), implying that these portions of GvpC did not interact with GvpA. The results confirmed the data obtained by pulldown assays described above. However, GvpN and GvpO bound Cterm of GvpC (rf 10–16), whereas the rf values observed with the N-terminal fragment Nterm were low (Figure 3E). Overall, our studies implied dimer formation (C/C, N/N, O/O), as well as the N/O, C/N, and C/O interactions, and the GvpN and GvpO interactions were confined to the C-terminal domain of GvpC (Figure 3F).

### Interaction of GvpA fragments with GvpA, C, N, and O

**Figure 4:**
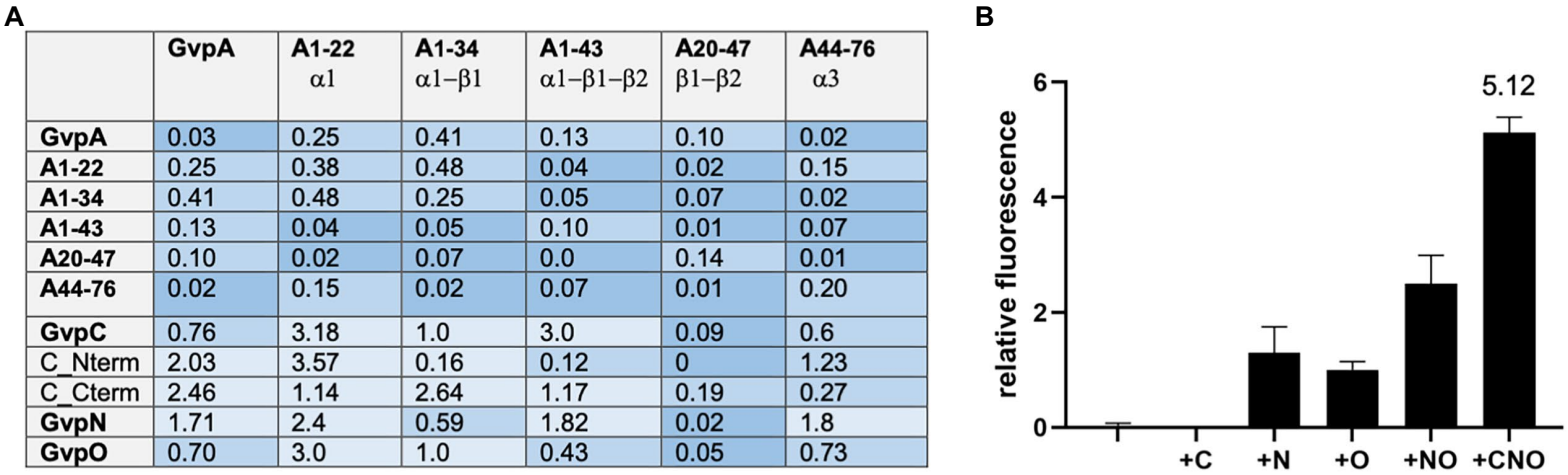
Interaction of the A fragments and dimerization of GvpA in presence of C, N, and/or O. The relative fluorescence (rf) obtained by split-GFP analyses is given in LAU/mm^2^. In each case, only the combination with the highest rf value is presented. **(A)** rf values obtained for the interactions of the different A fragments with each other, and also with GvpA, C, N, O, or the Nterm and Cterm fragments of GvpC. **(B)** Dimerization of GvpA in the presence of GvpC, N, and/or O.

Since the A/A interaction and an interaction of the entire GvpA with other Gvp proteins was difficult to detect by split-GFP analysis, we tested several fragments of GvpA encompassing different structural elements of GvpA (Völkner et al., 2020). Fragment A1-22 contains the first 22 aa including α-helix 1 (α1), fragment A1-34 contains α1 plus β-sheet 1 (α1-β1), fragment A1-43 consists of α1-β1-β2, fragment A20-47 of the two β-sheets β1-β2, and A44-76 contains mainly helix α3 of GvpA (Völkner et al., 2020; Jost et al., 2021). These GvpA fragments have been tested successfully with the GvpA-interaction partner GvpF. We used these GvpA fragments to analyze the GvpA dimerization, and also the interaction of GvpC, N and O in the respective *Hfx. volcanii* transformants. However, testing the different GvpA fragments against each other resulted in a very low fluorescence of rf ≤ 0.48 only (Figure 4A), indicating that the interaction sites of GvpA were difficult to determine by split GFP analysis. It is possible that the GvpA fragments exhibit an altered structure compared to GvpA molecules interacting in the shell. The highest rf value for the interactions of these A-fragments with GvpC, N, and O was always obtained with fragment A1-22 containing α1, and the highest fluorescence was observed with the C/A1-22 (rf 3.18) and O/A1-22 (rf 3.0) transformants (Figure 4A). Using the GvpC fragments Cterm and Nterm yielded the highest fluorescence with Nterm/A1-22 (rf 3.57), whereas the fluorescence obtained with all other fragments was low (rf < 1.23). In the case of Cterm, the highest fluorescence was observed with the Cterm/A1-34 transformant (rf 2.64; Figure 4A). Overall, it appears that the α-helical region of GvpC might contact GvpA, but the rf values are very low.

To investigate the influence of GvpC, GvpN and/or GvpO on the dimerization of GvpA, the A/A interaction was analyzed in the presence of one, two or all three of these (untagged) Gvp proteins. The respective *gvpC, gvpN,* and/or *gvpO* reading frame(s) were expressed under the control of the strong *P2* promoter in addition to a *gvpA* reading frame fused to *ngfp* expressed under *P_fdx_* promoter control in vector pJAS-NGFP-Nterm or pJAS-NGFP-Cterm. Both promotors insure a strong and constitutive expression during growth (Born and Pfeifer, 2019). The second *gvpA* reading frame fused to *cgfp* is present in vector pWL_fdx_-CGFP and expressed under *P_fdx_* control. The fluorescence determined for the resulting A/A, A/A + C, A/A + N, A/A + O, A/A + NO, and A/A + CNO transformants is shown in Figure 4B. A fluorescence of the A/A or A/A + C transformants was not detectable, and the fluorescence of the A/A + N, A/A + O or A/A + NO transformants was below rf 3. However, the A/A + CNO transformants exhibited the highest fluorescence (rf 5.12), implying that the presence of all three Gvp proteins supported the dimerization of GvpA and prevented at least some of the unspecific aggregations of this hydrophobic protein.

### Interaction of GvpA with variants of GvpF

Earlier studies by split-GFP determined GvpF as important interaction partner of GvpA (Völkner et al., 2020). The accessory protein GvpF is encoded by the first reading frame of the *gvpFGHIJKLM* mRNA expressed in early exponential growth. All of these accessory Gvp proteins are thus involved in early stages of gas-vesicle assembly (Offner and Pfeifer, 1995). Since GvpA fragment A1-22 yields a high fluorescence of F/A1-22 transformants (rf 42), we assumed that an interaction site of GvpF is located in helix α1 of GvpA (Völkner et al., 2020). The helix α1 of GvpA contains five charged aa (xxExxDRxxDK) that are all essential for gas vesicle formation; a substitution of any one of these aa results in Vac^−^ ∆A + A_mut_ transformants (Knitsch et al., 2017). To test whether the charged aa in GvpF form ion pairs with GvpA, single polar aa of GvpF was substituted by alanine to exclude the charge, or by other charged aa to reverse the charge. Glu or Asp was substituted by Arg or Lys, whereas a positively charged aa was always substituted by Glu (Supplementary Table S2). The different GvpF_mut_ variants were investigated in ∆F + F_mut_ transformants for the production of gas vesicles, and also by split-GFP for their interaction with GvpA in F_mut_/A transformants.

The ∆F construct was constructed by Gibson assembly (Gibson et al., 2009, 2011). ∆F contains except for *gvpF* all *gvp* genes of the p-vac region inserted in pWL102, and ∆F is complemented by *gvpF* or *gvpF*_mut_ inserted in pJAS35 (F or F_mut_ construct) in ∆F + F-or ∆F + F_mut_ transformants. Lysates of the transformants were inspected for the presence of GvpF or GvpF_mut_ by Western analysis using the GvpF antiserum (Supplementary Figure S1). In each case, the GvpF protein was well detectable. The possession of gas vesicles was tested after 5 weeks of growth of the ∆F + F_mut_ transformants on solid media by inspecting the colonies formed, and isolated gas vesicles were analyzed by electron microscopy. The phenotype of the ∆F + F transformants used as a control was turbid and pink, indicating the possession of gas vesicles (Vac^+^ phenotype; Supplementary Figure S2). The results on the respective ∆F + F_mut_ transformants are summarized in Supplementary Table S2. Most of the single aa alterations in GvpF yielded Vac^+^ ∆F + F_mut_ transformants, and the amount and shapes of the isolated gas vesicles were often similar to the wild type (Supplementary Figure S2). The four Vac^−^ ∆F + F_mut_ transformants obtained produced the GvpF variants E71R, E72A, E72R, or R213E. The residues E71 and E72 are not conserved between the GvpF proteins derived from the p-vac or c-vac region of *Hbt. salinarum*, or the mc-vac region of *Hfx. mediterranei*, whereas R213 is the very last aa and conserved in all three GvpF sequences. Smaller gas vesicles were obtained with the GvpF variants E03A, D15R, D19A, and D19R, exhibiting an average width of 100 nm compared to the 250 nm of the wild type (Supplementary Table S2). Longer, cylinder-shaped gas vesicles with small diameters were observed with the GvpF variants E14A and E65A, and longer, cylinder-shaped gas vesicles with a similar diameter as wild type with 10 GvpF variants (D15A, D53R, E54A, E57A/R, K68E, E70A, E71A, R73A, and K85A; Supplementary Figure S3; Supplementary Table S2). All these residues are located at the surface of GvpF and some of them are close to each other. All other transformants contained gas vesicles similar to wild type.

**Figure 5:**
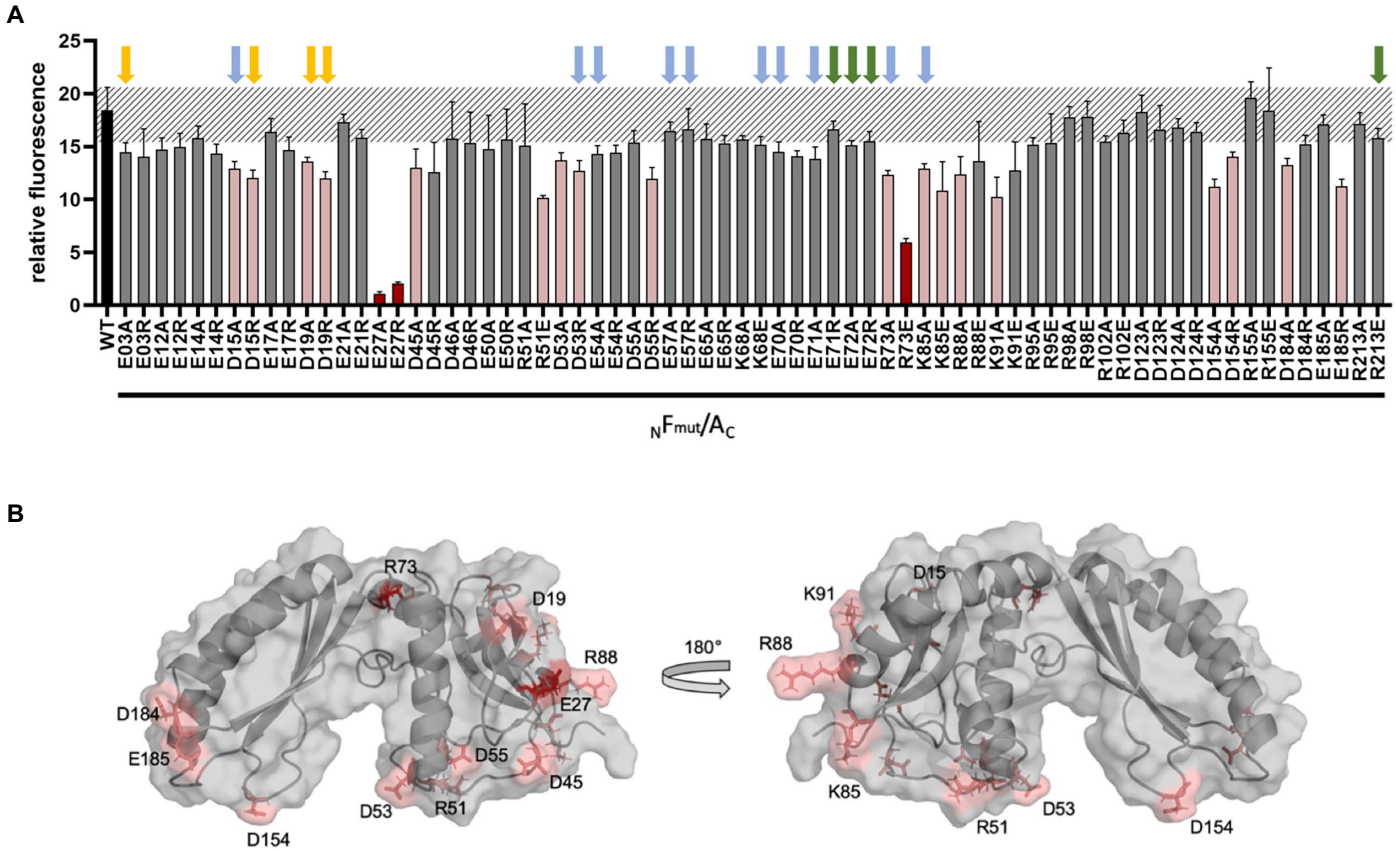
Split-GFP analysis of GvpF and of GvpF variants with GvpA. **(A)** The combination _N_F_mut_/A_C_ contains NGFP fused to the N-terminus of GvpF and CGFP to the C-terminus of GvpA. The respective substitution present in Fmut is indicated at the bottom of the graph. The relative fluorescence is given in LAU/mm^2^, and only the combination with the highest rf value is presented. The relative fluorescence was calculated in relation to the fluorescence of *Hfx. volcanii* wild type. The error bars represent the standard deviation of the rf values of the biological/technical replicates. The relative fluorescence obtained with the GvpF wild type and GvpA (WT) is labeled in black; the standard deviation determined for this wild type interaction is marked in grey stripes. The GvpF variants resulting in a strong reduction of the fluorescence in F_mut_/A transformants are marked in red, and pink indicates F_mut_/A transformants resulting in a smaller reduction of the fluorescence (value of *p* red ≤0.001; pink ≤0.05). Three biological and three technical replicates (*n* = 9) were performed in each case. Arrows above the graph indicate the presence or absence of gas vesicles in the respective ∆F + F_mut_ transformants. Colors used are: green, Vac^−^ transformants; yellow, small gas vesicles; blue, cylinder-shaped gas vesicles (also see Supplementary Table S2). **(B)** Homology model of GvpF and location of the substitutions leading to a reduction of the F_mut_/A interaction. The aa substitutions leading to a strong reduction are indicated in red, and a less reduced fluorescence in pink. The homology modeling based on the crystal structure of GvpF of *Microcystis aeruginosa* (Yang et al., 2015) was done using I-TASSER server.

The different GvpF variants were also studied in F_mut_/A transformants to determine their interaction potential with GvpA. GvpF is the only accessory Gvp where an interaction with GvpA is detectable by split-GFP analysis (Völkner et al., 2020; Jost et al., 2021). The F_wt_/A transformants indicated a high fluorescence value (rf 18; Figure 5A). Many of the GvpF variants yielded a similarly high fluorescence, suggesting that the particular substitutions in GvpF had no influence on the F_mut_/A interaction. Three GvpF variants, E27A, E27R and R73E, yielded a reduced fluorescence (rf 1.1–rf 5.9; Figure 5A, marked in red). The location of E27 and R73 in the 3D-model of GvpF is shown in Figure 5B. The homology model of GvpF was obtained using the crystal structure of a cyanobacterial GvpF protein (Yang et al., 2015) as a template for homology modeling. GvpF contains two domains, and E73 is located close to the region separating the two domains. The E27 residue is found at the surface of domain 1 (Figure 5B, left). However, none of these GvpF substitution variants resulted in a Vac^−^ phenotype (Supplementary Table S2), only the ∆F + F_mut_ transformants carrying an alteration of GvpF in E71 or E72 were Vac^−^. The substitution of several other amino acids yielded a slightly lower fluorescence of the F_mut_/A transformants (Figure 5A, marked in pink). The lowest fluorescence was observed with alterations of E27A/R and R73E, and some other GvpF variants indicated a lower fluorescence when substituted by alanine and by a reversal of the aa charge (variants D15A/R, D19A/R, D53A/R, R73A, K85A/E, and D154A/R; Figure 5A). Except for D53A, K85E, and D154A/R that contained gas vesicles of wild-type shape, these alterations resulted in smaller or longer gas vesicles. They are all located at the surface of GvpF (Figure 5B) and might mediate the A/F interaction (Supplementary Table S2). However, none of the Vac^−^ phenotypes correlated with a reduced fluorescence; the effects on the F/A interaction were thus not severe enough to affect gas vesicle formation.

### Interaction of GvpA, C, N, O with GvpF through GvpM

**Figure 6:**
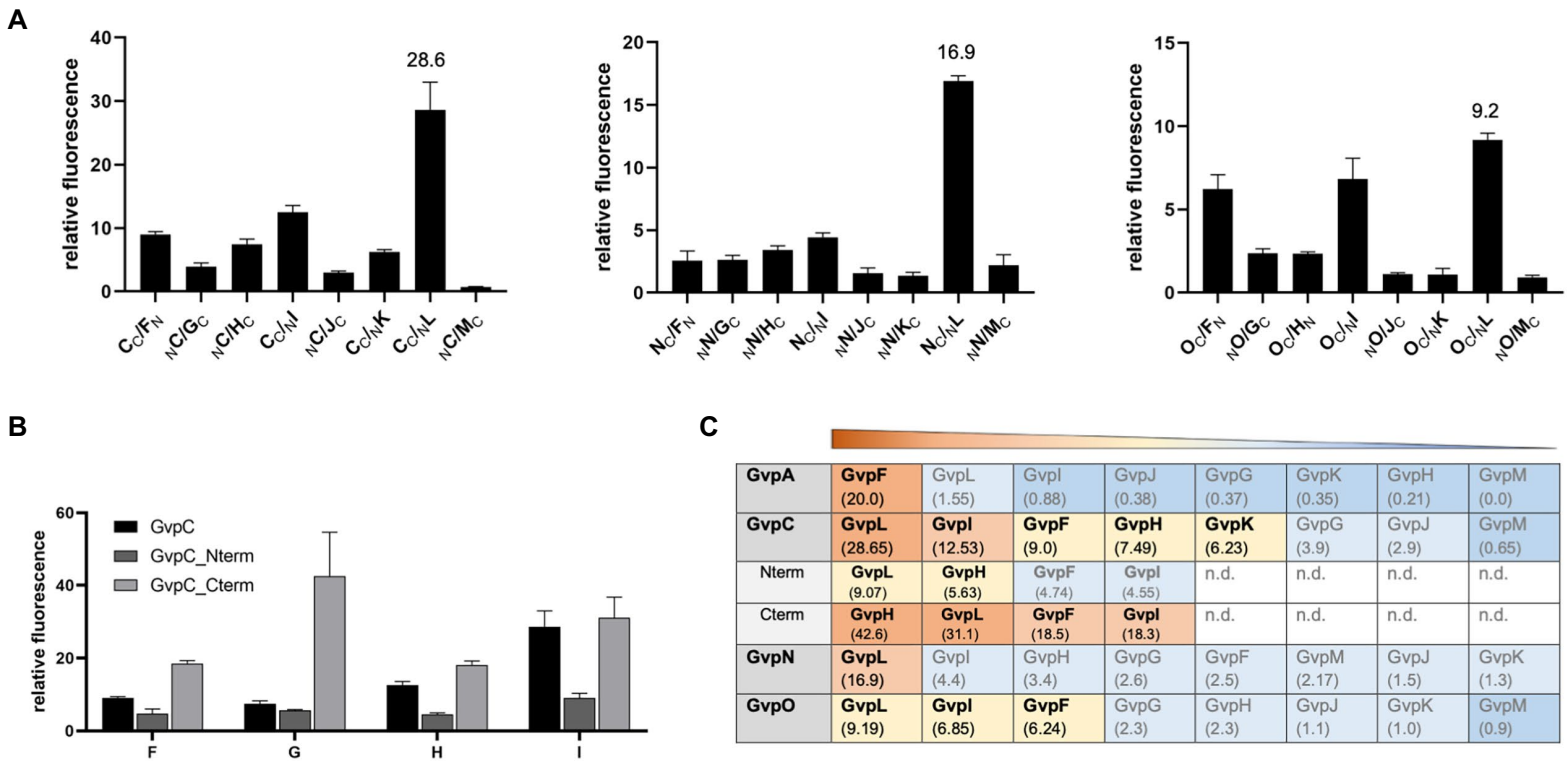
Spilt-GFP analysis of GvpC, N, and O with GvpF through GvpM. **(A)** The three graphs show the relative fluorescence obtained with C/−, N/−, and O/F through M transformants. **(B)** Relative fluorescence of GvpC or GvpC fragments Cterm and Nterm with GvpF, H, I, or L. **(C)** Overview of the rf values obtained for each of these interactions. The interacting Gvp proteins are ordered by rf values and shaded in red, rf > 10; yellow, rf < 10 to >5; blue, rf < 5; or dark blue, rf < 1; n.d., not determined.

GvpC, GvpN, and GvpO are hydrophilic proteins and were tested by split-GFP for their ability to interact with the accessory proteins GvpF through GvpM. In each case, only the combination yielding the strongest rf value of the eight combinations of NGFP- or CGFP-fusions tested is presented (Figure 6). GvpC interacted with GvpF, H, I, and L; the highest relative fluorescence was observed for the C/L transformants (rf 28.6), but also C/I, C/H, and C/F transformants yielded values above rf 7.5 (Figure 6A). GvpF, H, I, and L are thus able to interact with GvpC. GvpN interacted with GvpL only (rf 16.9); all other accessory Gvp tested yielded a fluorescence below rf 5. GvpO interacted with GvpF, I, and L (Figure 6A). Also, the two fragments of GvpC, Nterm and Cterm, were investigated to define the binding sites of the GvpF, H, I, and L more precisely. The transformants containing Cterm yielded the highest fluorescence in all cases, implying that the C-terminal portion of GvpC is able to contact all four proteins (Figure 6B). The fluorescence of the H/Cterm transformants was with rf 42.6 very high, but also the other three proteins yielded values above rf 18. The N-terminal GvpC fragment Nterm attracted GvpL and GvpH, but much lower rf values were observed. An overview of these results is shown in Figure 6C. Overall it became clear that the gas vesicle structural protein GvpC also binds accessory proteins derived from the leftward *gvp* operon, and that GvpL is able to bind any of the Gvp proteins tested except for GvpA.

**Figure 7:**
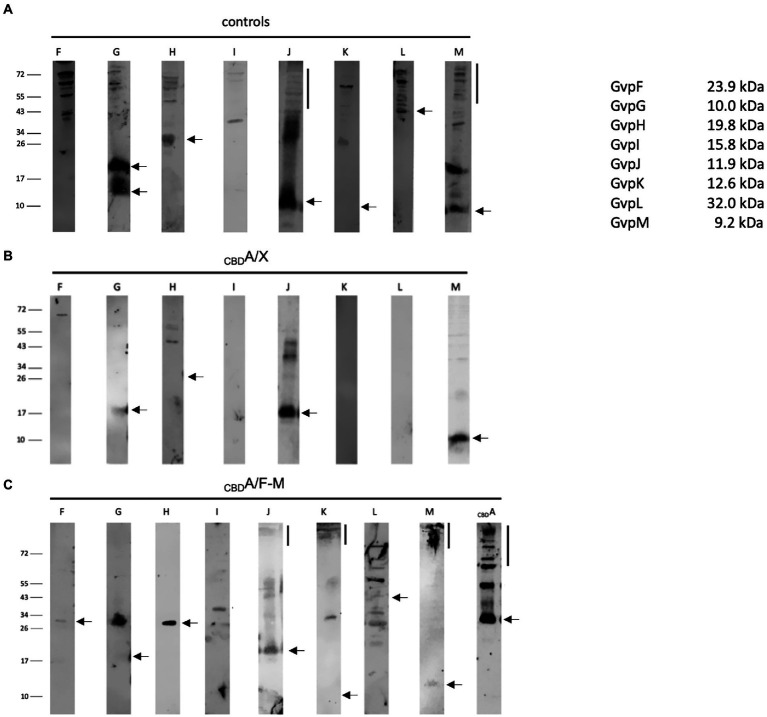
Western analyses of the pulldown assays using _CBD_A as bait and GvpF through GvpM as single proteins or altogether as prey. In each case, 15 μl of the elution sample was separated by SDS-PAGE, and the proteins were transferred to a PVDF membrane that was incubated with the respective antiserum. The proteins were visualized by the fluorophore-labeled secondary antibody IRDye 800 CW (Licor). The blots are inverted to black–white. Numbers on the left indicate sizes in kDa. **(A)** Western analysis of *Hfx. volcanii* producing the GvpF, G, H, I, J, K, L, or M protein used as control. The respective Gvp proteins are marked on top. **(B)** Results of the _CBD_A/X interactions (X = F, G, H, I, J, K, L, or M). The single Gvp protein used as prey is indicated on top. Only the elution fraction is shown. **(C)** Results on the _CBD_A/F-M interaction where GvpF through GvpM proteins is produced together in the same cell. Only the elution fraction is shown. The antiserum used to detect one of these Gvp proteins is indicated on top.

The interactions of the hydrophobic GvpA with all these accessory Gvp were analyzed by pulldown assays using _CBD_A as bait and GvpF through GvpM as prey. These proteins were tested either individually in _CBD_A/X transformants (X = F, G, H, I, J, K, L, or M), or altogether in _CBD_A/F-M transformants producing _CBD_A and GvpF through GvpM at the same time. In each case, _CBD_A plus putative interaction partner were eluted and separated by SDS-PAGE followed by Western analysis with the respective antiserum. The transformants producing GvpX proteins by themselves were used as control (Figure 7A). In the case of the _CBD_A/X transformants (X = F, G, H, I, J, K, L, or M), _CBD_A not only bound GvpF, but also GvpG, H, J, or M. The latter proteins yielded similar protein bands as observed in the controls (Figures 7A,B). The additional larger bands found with GvpJ also occurred in the control panel and are due to oligomers of this hydrophobic protein (Tavlaridou et al., 2014). In contrast, GvpI, K, and L were not selected by _CBD_A suggesting that these proteins are not able to interact with GvpA (Figure 7B). In the case of the _CBD_A/F-M transformants producing all accessory proteins at once, any of the accessory Gvp was pulled down by _CBD_A (Figure 7C). In particular, GvpI, K, and L were detected, implying that these Gvp proteins bound _CBD_A in complex with (an)other Gvp(s) partner. Several protein bands were detected close to the top of the separating gel (>150 kDa) in the elution fraction of the _CBD_A/F-M transformants probed with the J-, K-, M-, or A antiserum. Such large protein bands might indicate the formation of larger complexes and multimers of these Gvp proteins.

## Discussion

Studies on the interactions of Gvp proteins involved in gas-vesicle assembly are important to unravel the formation of these nanostructures. The eight accessory proteins GvpF through GvpM all interact and presumably form (a) complex(es) at the initial stage(s) or gas-vesicle assembly (Völkner et al., 2020; Jost et al., 2021). Here, we studied the proteins encoded by the *gvpACNO* operon and investigated their interactions using two different methods, split-GFP analysis and pulldown assays with CBD-tagged proteins.

### GvpN and GvpO form homo-and heterodimers and interact with GvpA and GvpC

The AAA-ATPase GvpN contains a Walker motif and hydrolyzes ATP as shown for the related cyanobacterial GvpN (Cai et al., 2020), whereas the function of the essential GvpO is not yet clear. Both Gvp proteins formed a homodimer as well as a heterodimer when analyzed by split-GFP (Figure 3). GvpN and GvpO bound to the conserved C-terminal domain of GvpC, implying a location at the exterior surface of the gas vesicles where GvpC stabilizes the gas vesicle shell formed by GvpA. However, GvpN is produced in much lower amounts compared to GvpA and GvpC, and the protein might be only locally present at the gas vesicle, whereas GvpC has been shown to cover the entire gas vesicle surface (Kinsman et al., 1995; Dunton et al., 2006). Our pulldown assays suggested that GvpN and GvpO interact with GvpA, and monomers as well as multimers of GvpA were selected especially by _CBD_O (Figure 2). Thus, in addition to GvpF and GvpJ (Völkner et al., 2020; Jost et al., 2021), also GvpN and GvpO are able to contact GvpA. The AAA-ATPase GvpN might promote the incorporation of GvpA during early stages of the gas-vesicle assembly, since only small gas vesicles are formed in ∆N transformants lacking GvpN (Offner et al., 1996). GvpO could be a bridging protein connecting GvpN-GvpA or GvpC and GvpA. However, these assumptions should be tested in more detail.

### An interaction of GvpA and GvpC was not observed

While _CBD_N/A and _CBD_O/A interactions were well detectable despite the hydrophobic character of GvpA, _CBD_C did not interact with GvpA, and vice versa, _CBD_A did not select GvpC in pulldown assays (Figure 2). Also, any of our split-GFP analyses performed to detect the A/C interaction was not successful, even when fragments of both proteins were applied (Figure 3). This was surprising since GvpC adheres at the surface of the gas vesicles as shown by tryptic digests of isolated cyanobacterial gas vesicles of *Anabaena flos-aquae* and identification of the GvpC fragments still bound (Kinsman et al., 1995; Dunton et al., 2006). These fragments can be removed from isolated gas vesicles by rinsing in 6 M urea without the structure collapsing, and an investigation by MALDI-TOF mass spectrometry yielded that the GvpC fragments consist of both ends of the 33-residue repeats. In the case of the haloarchaeal GvpC, the protein is rinsed of gas vesicles with water (Englert and Pfeifer, 1993; Knitsch et al., 2017). The N-terminal fragment Nterm used for split-GFP analyzes in this report consists of three of the seven α-helical repeats of GvpC, but the fluorescence obtained for Nterm/A1-22 transformants was indicative of a weak interaction only (Figure 4A). An even lower rf value was determined for Cterm/A1-22 transformants. It is possible that the N-terminal GvpA region mediates the A/C contact *via* the α-helices of GvpC as observed for cyanobacterial gas vesicles (Belenky et al., 2004; Dunton et al., 2006). In favor of the assumption that A1-22 of GvpA is involved was that the highest rf values are always detected when N-or CGFP was fused to the C-terminus of GvpA (Figure 3A); an N-terminal fusion might interfere with the GvpC interaction. Along this line, the barely detectable _CBD_A/C interaction might be due to a reduced accessibility of the N-terminal region of GvpA fused to CBD.

The trypsin treatment of the gas vesicles of *A. flos-aquae* rinsed with concentrated urea to remove the adhering GvpC cleaves GvpA near the N-terminus and results in the tetrapeptide AVEK of GvpA (Belenky et al., 2004). A similar result has been described by Dunton et al. (2006), and the authors also identified the adjacent peptide TNSSSLAEVIDR after tryptic digest of gas vesicles, but this fragment was not released. Other putative trypsin sites in GvpA are protected (Dunton et al., 2006). The aa sequence LAEVIDR is part of helix α1 of GvpA and also conserved in the haloarchaeal GvpA. The results suggest that helix α1 is located at the exterior surface of the gas vesicle, supporting the idea that GvpC contacts this region. Another explanation for the lack of a GvpA-GvpC interaction is that GvpC might only interact with GvpA molecules assembled in the gas vesicle shell. Walsby (1994) calculated a ratio of one GvpC to 25 GvpA molecules and suggested that each of the helices of GvpC might span five GvpA molecules per rib. The five α-helical repeats of the *Anabaena* GvpC would then span five adjacent ribs to stabilize the shell.

### A/A interactions were not observed, but A/F interactions were well detectable

The interaction of two GvpA monomers was not observed by split-GFP, presumably due to the hydrophobic character of GvpA leading to unspecific GvpA aggregates in the solution. To prevent these aggregations, five different fragments of GvpA were applied. Fragments A1-22, A1-34, and A1-43 of GvpA were already successfully used to determine the interaction of GvpA with GvpF more precisely, but GvpF also binds the entire GvpA (Völkner et al., 2020). In contrast, fragments A20-47 and A44-76 did not interact with GvpF. To determine putative A/A interaction sites, the different combinations of the A fragments were investigated by split-GFP. However, none of these combinations resulted in a detectable fluorescence of the respective transformants (Figure 4A). It is possible that the 3D-structure of the A fragments differs from the 3D-structure of GvpA when assembled in the gas vesicle shell, or that the interaction sites are not properly formed in the fragments. The highest rf values were always determined with A1-22 (rf 3.0–3.5) that also interacted with GvpF, C, N, or O. Most likely, the 22-aa region of A1-22 is too small to bind all of these Gvp proteins simultaneously; the interactions might thus occur sequentially, or either occur at the stage of a GvpA monomer, or of GvpA aggregated in the shell. The different Gvp proteins might also bind at different locations of the gas vesicle to GvpA. The putative functions of these interaction partners are quite different; GvpF produced in the early growth stage is a constituent of the nucleation complex formed early in gas-vesicle assembly; the protein is able to bind GvpA as a monomer (Völkner et al., 2020). GvpC is produced throughout growth and adheres to the gas vesicle surface from the very beginning, whereas GvpN hydrolyzes ATP and might be required for the incorporation of GvpA into the shell to enlarge the gas vesicles. GvpO might connect GvpA, C, and/or N. It is interesting to note that the dimerization of GvpA was enhanced in the presence of the three proteins GvpC, N, and O. Especially, GvpC was important; GvpN, O, or N/O were not sufficient to support a well detectable GvpA dimerization (Figure 4B). Keeping this in mind, it is surprising that ∆C transformants (lacking GvpC but containing all other Gvp) are Vac^+^ and produce gas vesicles. However, these are less stable and exhibit unregular shapes, suggesting that GvpC is important to yield the original spindle or cylinder shape of the gas vesicles (Offner et al., 1996).

Earlier split-GFP analyses determined fragment A1-22 as a major contact site of GvpF and the residues R15, K19, and G20 as important for the interaction (Völkner et al., 2020). Since two of the charged aa of α1 are involved in the A/F interaction, we tested whether polar aa of GvpF might form ion pairs with GvpA, and are required for the A/F interaction and/or gas vesicle formation. Single substitutions of D-, E-, R-, or K residues of GvpF by alanine or oppositely charged aa were tested by split-GFP analyses in A/F_mut_ transformants, and the GvpF variants were also analyzed in ∆F + F_mut_ transformants for their ability to support gas vesicle formation. Only a few of these substitutions (E27A, E27R, R73E) resulted in a strong reduction of the fluorescence of A/F_mut_ transformants, and these aa are distributed on the surface of GvpF (Figure 5B). However, a correlation between a reduced A/F interaction and a Vac^−^ phenotype. Thus, none of the aa in GvpF altered to prevent putative ion bonds was important for the F/A interaction was not observed (Figure 5; Supplementary Table S2). Thus, the aa in GvpF altered to prevent putative ion bonds was important for the F/A interaction. Some of the substitutions in GvpF resulted in an altered shape of the gas vesicles (smaller or longer cylinder-shaped ones) in ∆F + F_mut_ transformants. Since GvpL, G, H, I, C, and O also interact with GvpF, one or more of these other interactions could be affected in Vac-transformants.

### GvpC, N, and O bind other accessory Gvp

Several additional interaction partners were determined for GvpC (GvpF, H, I, K, and L) and GvpO (GvpF, I, L), whereas GvpN bound GvpL only (Figure 6). The latter result showed that GvpL is able to bind except for GvpA all of the Gvp proteins (Völkner et al., 2020, and this report). The highest rf values were found for F/−, H/−, I/−, and L/Cterm transformants indicating that these proteins mainly bound to the C-terminal domain of GvpC, similar to GvpN and GvpO, whereas GvpA might prefer to bind the N-terminal fragment of GvpC. The interactions with GvpF, H, I, K, and L could only occur in early stages of growth when these proteins are produced, whereas GvpN and GvpO might also interact in later growth stages. The initiation complex formed by the accessory Gvp proteins could be an anchor for GvpA and also for GvpC at the start to form the gas vesicle caps.

GvpC was found to dimerize *via* Cterm/Cterm and Nterm/Cterm interactions, whereas the Nterm/Nterm interactions were not detectable (Figure 3E). A GvpC dimer is also the major protein band observed when GvpC is produced by itself in C transformants (Figure 2A). GvpC might form a long α-helical rod consisting of the related repeats. The interactions between the C- and N-terminus (Cterm/Cterm and Cterm/Nterm) of GvpC might enable the formation of a larger network of GvpC molecules. GvpN and GvpO bound to the C-terminal region of GvpC and could compete with the Cterm/Cterm interaction and thus with the dimerization or polymerization of GvpC. The pulldown assays with _CBD_N/C or _CBD_O/C yielded a GvpC monomer but not the dimer, suggesting that GvpN and GvpO were able to interfere with the dimerization of GvpC (Figure 2B). It might be important to prevent the C/C interaction during gas vesicle formation before GvpC adheres at the exterior surface. Preventing the C/C interaction might be also important in the area where GvpA is incorporated to enlarge the gas vesicle shell, a location where also the action of GvpN is required. We presume that the enlargement of the two helical ribs forming the left and right portion of each gas vesicle occurs at the center of the spindle or cylinder structure.

### _CBD_A interacts with GvpF, G, H, J, and M, but not with GvpI, K and L

The interaction of GvpA with GvpF through GvpM has been already studied by split-GFP analyses, and GvpF and GvpJ were identified as interaction partners (Völkner et al., 2020; Jost et al., 2021). Here, we performed pulldown assays with _CBD_A/X transformants (X = F, G, H, I, J, K, L, or M) as prey to analyze each interaction directly, but also with _CBD_A/F-M transformants synthesizing _CBD_A and GvpF through GvpM altogether in the same cell. Testing the single accessory Gvp, _CBD_A selected GvpF, G, H, J, and M, but not GvpI, K, and L (Figure 7B). However, all accessory Gvp were selected in _CBD_A/F-M transformants, demonstrating that other interaction partner(s) enabled the pulldown of GvpI, K, and L by _CBD_A (Figure 7C). Multimers of _CBD_A up to the top of the separating gel were detected in the Western analysis of the _CBD_A/F-M lysate, and antisera raised against GvpJ, K, and M reacted with bands of similarly large sizes, implying that these proteins are either part of the GvpA aggregate or formed large multimers by themselves. Multimer formation has been already observed for the hydrophobic GvpJ and GvpM exhibiting sequence similarities to GvpA (Tavlaridou et al., 2014; Jost et al., 2021), and with the hydrophilic GvpK in the presence of GvpI (Völkner et al., 2020). The patterns of GvpJ oligomers were very similar in lysates of _CBD_A/F-M and _CBD_A/J transformants, but larger compared to the GvpJ control (Figure 7). Different protein bands were observed with other accessory Gvp in _CBD_A/F-M-compared to _CBD_A/X transformants (Figures 7B,C). The larger bands found for GvpG and GvpM in _CBD_A/F-M transformants compared to the _CBD_A/G-or _CBD_A/M transformants might be due to protein complex(es) formed that are stable in SDS-containing buffers.

### Implications for gas vesicle formation

The accessory proteins GvpF through GvpM are produced, together with GvpA, C, N, and O, in minor amounts at an early stage of gas-vesicle assembly. Some or all of the GvpF through GvpM might form a nucleation complex and initiate the assembly of the shell. The major constituent (<95%) is the hydrophobic GvpA. GvpF, G, H, J, M as well as GvpN and O bind GvpA, whereas GvpI, K, and L do not interact directly (Table 2). Since GvpF and GvpO bound GvpA also as monomer, it is possible that they enable GvpA to stay soluble before being incorporated into the shell. GvpO also interacts with GvpF, and both interact with GvpL, the major platform for all other Gvp proteins. GvpL also attracts GvpC. The ATPase GvpN might assist in these processes; the protein could power the subunit-turnover and/or the GvpA incorporation in the shell. Most likely, gas-vesicle assembly starts at the tips of the conical cap structures, with GvpA forming a low-pitch helix of increasing diameters up to the final 200–250 nm in the cylinder portion (Offner et al., 1998). The accessory Gvp might help to initiate the rib-formation with increasing diameters in both caps. Studies on cyanobacterial gas vesicles determined GvpF at the gas-facing surface of the shell (Yang et al., 2015), and our previous analyses indicate that the residues R28 and E40 of GvpA are important for the GvpF interaction (Knitsch et al., 2017; Völkner et al., 2020). Solid-state NMR studies propose that these two amino acid residues also contribute to the A/A interaction in the ribs of the shell (Sivertsen et al., 2010; Bayro et al., 2012). The presence or absence of GvpF might determine whether the R28 and E40 form ion bonds in the A/F connection or support the A/A interaction in the shell. The presence of GvpF at the gas-facing surface might also prevent interactions of this hydrophobic surface, especially at the low distance close to the tips of the caps.

**Table 2:** Interaction of Gvp proteins as found by split-GFP analysis.

Gvp	Interaction partners^*^		Dimer/heterodimer formation^**^
	F, G, H, I, J, K, L, M	A, C, N, O	
A	F	N/O	A/A + CNO
A1–22	J1–56, J46–114		
C	L, H/I, F, K	N	C/C + multimers
Nterm			
Cterm	F, H/I, L	N/O	
N	L	C, O	N/N; N/O
O	L, F, I	N	O/O; N/O
F	L, H/I, G	A	
G	L, F		
H	L, F, I	H/I	
I	L, F, H	H/I	
J	L		
J1–56	G, H, L; M1–25, M60–84	A1–22	J/M, A/J
J46–114	L; M1–25, M60–84	A1–22	J/M, A/J
K	L		
L	G, F, H/I, J/M, K	C, N/O	
M	L		
M1–25	L; J1–56, J46–114		J/M
M60–84	L, F, H; J1–56, J46–114		J/M

* Summary of results from the current work and past studies (Völkner et al., 2020; Jost et al., 2021).

** Heterodimers found frequently are highlighted by colors: H/I, yellow; N/O, green; heterodimers of the AJM family, red.

Since the cyanobacterial GvpC covers the entire gas vesicle surface (Buchholz et al., 1993), GvpC needs to attach to this protein complex at the very beginning. Initial contacts of GvpC might be mediated by GvpF, H, I, K, and/or L, but GvpN and GvpO might also play a role. The repeating α-helices of GvpC might form a long rod-shaped protein, each of them spanning a rib formed by GvpA as suggested by Walsby (1994). A network of GvpC might be present at the surface, with interruptions by GvpN and/or GvpO bound at the C-terminal portion of GvpC. A crystal structure of GvpC and especially of the C-terminal globular portion might shed more light on the GvpC structure and the formation of GvpC dimers.

## Conclusion

Overall, eight of the 14 haloarchaeal *gvp* genes are essential (*gvpAO* and *gvpFGJKLM*), and many of these Gvp proteins are conserved between bacteria and haloarchaea underlining the importance of their function for gas vesicle formation in these microbes. The expression of the core genes *gvpA* and *gvpC* is not sufficient to allow the formation of functional gas vesicles in the original host. However, since gas vesicles are an attractive object for applications in biomedical research, the production of gas vesicles was tested in different fast-growing bacteria and eukaryotic cell cultures. The heterologous expression of the bacterial *gvp* genes has been achieved in other bacteria and in human cell lines, where the *gvp* gene cluster from *B. megaterium* (Li and Cannon, 1998) was successfully expressed (Farhadi et al., 2019). The accessory proteins GvpN, S, and U support abundant gas-vesicle assembly, whereas GvpF, G, J, K, and L (also essential for haloarchaeal gas vesicle formation) represent a “bottleneck” and have to be supplied in addition (Farhadi et al., 2019). Interestingly, GvpA and GvpC of *Planktothrix rubescens* are sufficient to form gas-filled nanoparticles in a human cell line (Mizushima et al., 2020), and both Gvp proteins of *Anabaena flos-aquae* form gas vesicles in *Saccharomyces cerevisiae* without the help of any additional accessory Gvp (Jung et al., 2021). This is surprising, since the different accessory Gvp proteins are required in the original host to form a large number of stable, gas-filled nanostructures. Endogenous chaperones present in eukaryotic cells might substitute for some of the accessory Gvp functions required in the original host. It would be rewarding to localize the different Gvp proteins at the gas vesicles during their formation, and determine whether they are only present in early stages or are permanent constituents of the gas vesicle shell. Such proteome analyses will require synchronized cells and a directed production of gas vesicles; cells grown in batch culture usually differ in age and each cell also contains a mixture of juvenile and older gas vesicles.

## Data availability statement

The original contributions presented in the study are included in the article/Supplementary material, further inquiries can be directed to the corresponding author.

## Author contributions

AJ and FP planned the study, discussed the results, and wrote the manuscript. AJ performed the analysis. All authors contributed to the article and approved the submitted version.

## Funding

This work was financially supported by the German Research Foundation, DFG (PF 165/15-1). We also acknowledge support by the German Research Foundation and the Open Access Publishing Fund of Technical University Darmstadt.

## Conflict of interest

The authors declare that the research was conducted in the absence of any commercial or financial relationships that could be construed as a potential conflict of interest.

## Publisher’s note

All claims expressed in this article are solely those of the authors and do not necessarily represent those of their affiliated organizations, or those of the publisher, the editors and the reviewers. Any product that may be evaluated in this article, or claim that may be made by its manufacturer, is not guaranteed or endorsed by the publisher.
