# Supplementary material for: Interaction of the gas vesicle proteins GvpA, GvpC, GvpN, and GvpO of Halobacterium salinarum
Source: Front Microbiol. 2022 Jul 29;13:971917. doi: 10.3389/fmicb.2022.971917 (PMC9372576; doi:10.3389/fmicb.2022.971917)
Supplement: Supplementary file 1 [file Data_Sheet_1.PDF]

Supplementary Figures and Tables

Interaction of the Gas Vesicle Proteins GvpA, GvpC, GvpN and GvpO of *Halobacterium salinarum*

***Alisa Jost*[1] *and Felicitas Pfeifer*[1]**

[1]*Microbiology and Archaea, Department of Biology, Technical University Darmstadt, Darmstadt, Germany*

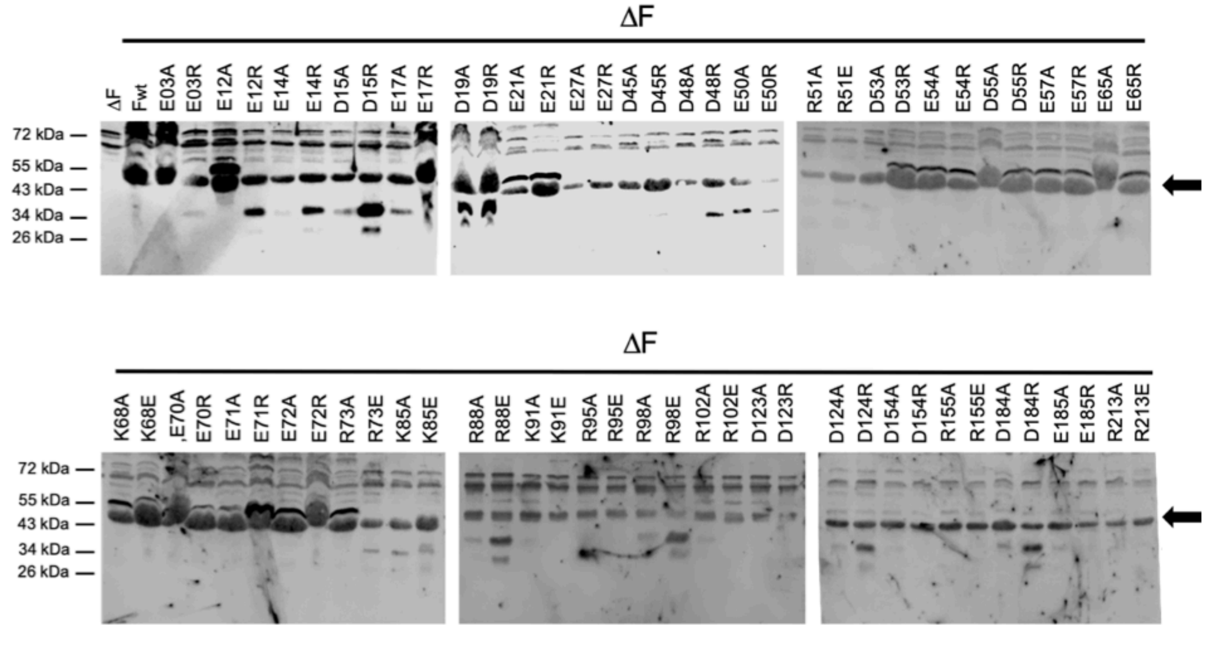

Supplementary Figure S1. Western analysis to determine GvpF in $\Delta F+F_{mut}$ transformants. Total proteins were isolated from $\Delta F+F_{wt}$ or $\Delta F+F_{mut}$ transformants in late exponential growth phase, and 20 µg of proteins were separated by SDS-PAGE, followed by transfer on a PVDF membrane and treatment with an antiserum raised against GvpF. The respective substitution- or deletion variant is indicated on top. Arrows on the right mark the position of GvpF.

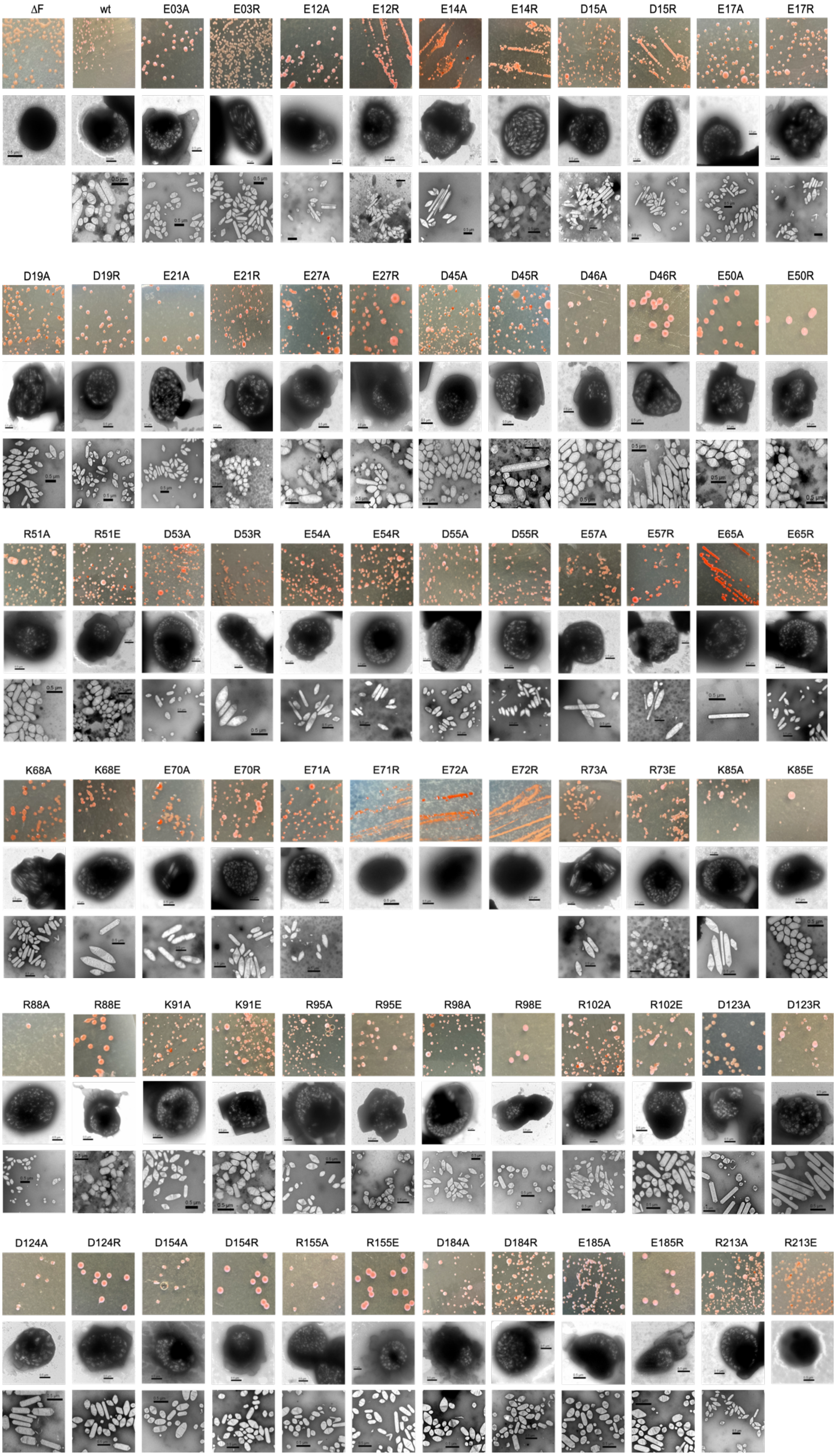
ΔF
wt
E03A
E03R
E12A
E12R
E14A
E14R
D15A
D15R
E17A
E17R
D19A
D19R
E21A
E21R
E27A
E27R
D45A
D45R
D46A
D46R
E50A
E50R
R51A
R51E
D53A
D53R
E54A
E54R
D55A
D55R
E57A
E57R
E65A
E65R
K68A
K68E
E70A
E70R
E71A
E71R
E72A
E72R
R73A
R73E
K85A
K85E
R88A
R88E
K91A
K91E
R95A
R95E
R98A
R98E
R102A
R102E
D123A
D123R
D124A
D124R
D154A
D154R
R155A
R155E
D184A
D184R
E185A
E185R
R213A
R213E

Supplementary Figure S2 (previous page). Colonies of $\Delta F+F_{wt}$ and $\Delta F+F_{mut}$ transformants on solid media (top), cells analyzed by transmission electron microscopy (TEM) for the possession of gas vesicles (middle), and isolated gas vesicles analyzed by TEM. The respective GvpF variants tested are indicated on top. The bar equals 0.5 µm in each case. Further explanations are given in the text.

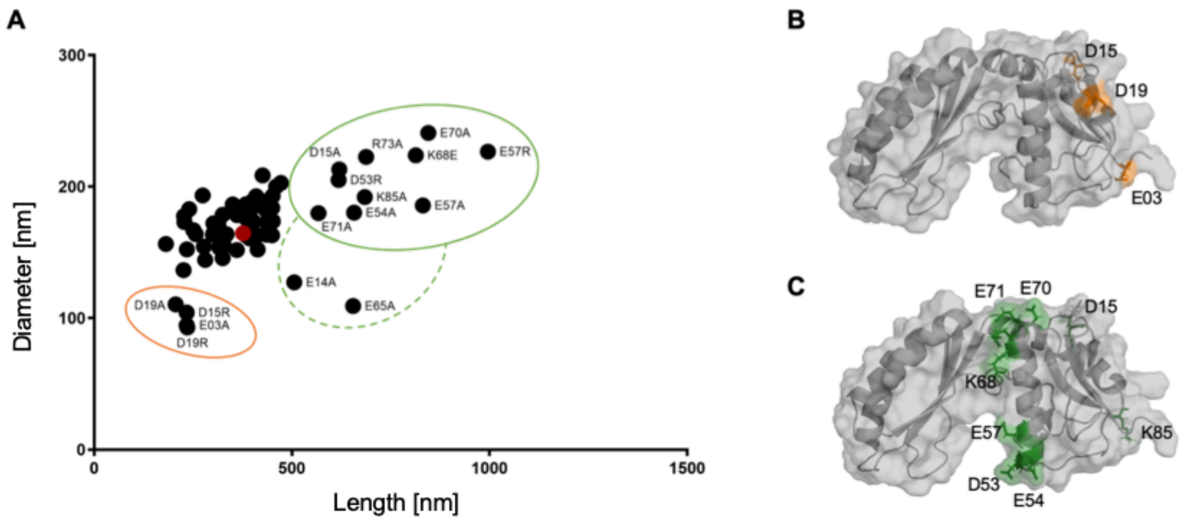

Supplementary Figure S3. Analysis of the morphology of gas vesicles derived from $\Delta F+F_{mut}$ transformants. **(A)** Scatter plot of gas vesicles isolated from $\Delta F+F_{mut}$ transformants depending on the length and the diameters of gas vesicles. Approximately 100 gas vesicles were measured with ImageJ and the median was reported. The value determined for wild type gas vesicles is labelled in red. The cluster of shorter gas vesicles is circled in orange; transformants containing longer gas vesicles in green (dashed line: smaller diameter). **(B, C)** Homology model of GvpF based on the crystal structure of the cyanobacterial GvpF (Xu et al., 2014) and modelled using I-TASSER Server (Zang, 2008; Roy et al., 2010; Yang et al., 2015). Amino acid substitutions leading to smaller gas vesicles are labelled in orange **(B)**, and aa substitutions leading to longer gas vesicles are labelled in green **(C)**.

Supplementary Table S1. Oligonucleotides used in these studies.

Name	Sequenz (5‘ → 3‘)
**ΔF construct**	
Δ_pWL102_fwd	aattacagtcggtcgggttcgagtcggagcCTACAGTTCCTCTTTCATG
ΔF_rv	gcctccttgttgctgCTCAGTCATTGGTCTCTC
ΔF_fwd	agaccaatgactgagCAGCAACAAGGAGGCCGATAATG
Δ_pWL102_rv	gttcctggccttttgctggccttttgctcaTCAGTCCTCTCGCCGATC
**GvpF substitutions**	
f-pF/E03A	ACT**gcg**AACCTATACACATACG
f-pF/E03R	ACT**cgg**AACCTATACACATACG
r-pF/E03	CATCTGCAGCCCGG
f-pF/E12A	GTATCATC**gca**CAGGAAGATCTCGAATTAG
f-pF/E12R	GTATCATC**aga**CAGGAAGATCTCGAATTAG
f-pF/E14A	GTATCATCGAACAG**gca**GATCTCGAATTAG
f-pF/E14R	GTATCATCGAACAG**aga**GATCTCGAATTAG
f-pF/D15A	GTATCATCGAACAGGAA**gca**CTCGAATTAG
f-pF/D15R	GTATCATCGAACAGGAA**aga**CTCGAATTAG
r-pF/E12+E14+ D15	CGTATGTGTATAGGTTCTCAGTCAT
f-pF/E17A	GATCTC**gca**TTAGATGTCGAAGGCGTTG
f-pF/E17R	GATCTC**aga**TTAGATGTCGAAGGCGTTG
f-pF/D19A	GATCTCGAATTA**gca**GTCGAAGGCGTTG
f-pF/D19R	GATCTCGAATTA**aga**GTCGAAGGCGTTG
f-pF/E21A	GATCTCGAATTAGATGTC**gca**GGCGTTG
f-pF/E21R	GATCTCGAATTAGATGTC**aga**GGCGTTG
r-pF/E17+D19+ E21	TTCCTGTTCGATGATACCGTATGTGTATAG
f-pF/E27A	GGAGCG**gca**CAGGTCTATC
f-pF/E27R	GGAGCG**cga**CAGGTCTATC
r-pF/E27	GGCAACGCCTTCGACATCTAATTC
f-pF/D45A	ACATT**gct**ACGACCGACCC
f-pF/D45R	ACATT**cgt**ACGACCGACCC
f-pF/D48A	ACATTGATACGACC**gca**CCCG
f-pF/D48R	ACATTGATACGACC**aga**CCCGAG
r-pF/D45+D48	CAGAGACGACAGCGGAGAG
f-pF/E50A	CCC**gca**CGCACCGATG
f-pF/E50R	CCC**cga**CGCACCGATG
f-pF/R51A	CCCGAG**gca**ACCGATG
f-pF/R51E	CCCGAG**gag**ACCGATGAG
r-pF/E50+R51	GTCGGTCGTATCAATGTCAGAGACGAC
f-pF/K85A	CGTTC**gca**AGTGCGCGCAC
f-pF/K85E	CGTTC**gaa**AGTGCGCGCAC
r-pF/K85	CCATCCCGAAGCTCATCGG
f-pF/R88A	GTGCG**gca**ACGCTAAAGGG
f-pF/R88E	GTGCG**gaa**ACGCTAAAGGG
r-pF/R88	TTTTGAACGCCATCCCGAAGC
f-pF/K91A	CACGCTA**gca**GGTGTATTG
f-pF/K91E	CACGCTA**gaa**GGTGTATTG
r-pF/K91	CGCGCACTTTTGAACG
f-pF/R95A	GTGTATTG**gca**GGGGCGC
f-pF/R95E	GTGTATTG**gaa**GGGGCGC
r-pF/R95	CCTTTAGCGTGCGCGC
f-pF/R98A	GGCG**gca**CGTGCATTG
f-pF/R98E	GGCG**gaa**CGTGCATTG
r-pF/R98	CCGCGCAATACACCCTTTAG
f-pF/R102A	CATTG**gca**AGTACGCTGAATGAC
f-pF/R102E	CATTG**gaa**AGTACGCTGAATGAC

r-pF/R102	CACGTCGCGCCCC
f-pF/D123A	CCTGGC**gca**GATACAGTCC
f-pF/D123R	CCTGGC**cga**GATACAGTCC
f-pF/D124A	CCTGGCGAC**gca**ACAGTCC
f-pF/D124R	CCTGGCGAC**cga**ACAGTCC
r-pF/D123+D124	ACCGAGTATCTTCACGCCAAGTTC
f-pF/D154A	CTTCACA**gca**CGCCTGATCATCAATAAG
f-pF/D154R	CTTCACA**cga**CGCCTGATCATCAATAAG
f-pF/R155A	CTTCACAGAC**gca**CTGATCATCAATAAG
f-pF/R155E	CTTCACAGAC**gag**CTGATCATCAATAAG
r-pF/D154+R155	AGATCGTTCTCGGTCTCGTTGATAC
f-pF/D184A	GGAATAC**gca**GAACTGACGATTCAG
f-pF/D184R	GGAATAC**cga**GAACTGACGATTCAG
f-pF/E185A	GGAATACGAC**gca**CTGACGATTCAG
f-pF/E185R	GGAATACGAC**cga**CTGACGATTCAG
r-pF/D184+E185	GCTTCGACATCGTCGATGGC
f-pF/R213A	TACCTTAT**gcg**CCTCCTTGTTG
f-pF/R213E	TACCTTAT**tcg**CCTCCTTGTTG
r-pF/R213	CCCAATTCGCCCTATAGTGAGTC

GvpF substitutions in split-GFP vectors

5'-BspHI-pF/E03A	attctcATGACTGCGAACCTATACACATACG
5'-BspHI-pF/E03R	attctcATGACTCGGAACCTATACACATACG
3'-BlpI-pF/R213A	attcgctcagcgaTTATGCGCCTCCTTGTTGCTGTTC
3'-BlpI-pF/R213E	attcgctcagcgaTTATTCGCCTCCTTGTTGCTGTTC
3'-KpnI-pF	attcggtaccTTATCGGCCTCCTTGTTGC
3'-KpnI-pF/R213A	attcggtaccTTATGCGCCTCCTTGTTGCTGTTC
3'-KpnI-pF/R213E	attcggtaccTTATTCGCCTCCTTGTTGCTGTTC

Split-GFP vectors

5'-BspHI-pC	attctcATGAGTACTGCCCGC
5'-BamHI-pC	attcggatccATGAGTACTGCCCGC
3'-BlpI-pC	attcgctcagcTCATGTTTTATCATCCGGCC
3'-BlpI-pCΔStop	attcgctcagcgaTGTTTTATCATCCGGCC
3'-BamHI-pCΔStop	agttctggatcccTGTTTTATCATCCGGCC
3'-BsrGI-pC	attctgtacatcATGTTTTATCATCCGG
5'-BspHI-pN	attctcATGACGAACGAGTCC
5'-BamHI-pN	attcggatccATGACGAACGAGTCCC
3'-BlpI-pN	attcgctcagcTTAAGAAAGGGCGACTTC
3'-BlpI-pNΔStop	attcgctcagcgaAGAAAGGGCGACTTC
3'-BamHI-pNΔStop	agttctggatcccAGAAAGGGCGACTTC
3'-KpnI-pN	attcggtaccTTAAGAAAGGGCGACTTC
5'-NcoI-pO	attcccATGGCAGATCCAGCAAA
5'-BamHI-pO	attcggatccATGGCAGATCCAGCAAAC
3'-BlpI-pO	attcgctcagcCTACAGTTCCTCTTTCATG
3'-BlpI-pOΔStop	attcgctcagcgaCAGTTCCTCTTTCATGTC
3'-BamHI-pOΔStop	agttctggatcccCAGTTCCTCTTTCATGTC
3'-KpnI-pO	attcggtaccCTACAGTTCCTCTTTCATGTC
3'-BlpI-pC_Nterm	attcgctcagctcaGTCGTCCGCATATGCTTC
3'-BlpI-pC_Nterm ΔStop	attcgctcagcgaGTCGTCCGCATATGCTTC
3'-BamHI-pC_NtermΔStop	attcggatcccGTCGTCCGCATATGCTTC
3'-KpnI-pC_Nterm	attcggtacctcaGTCGTCCGCATATGCTTC
5'-NcoI-pC_Cterm	attcccatgGAGACAGAGGAAGAGGC
5'-BamHI-pC_Cterm	attcggatccatgGAGACAGAGGAAG

P2-split-GFP vectors

Oligo_P2_antiparallel_fwd	gatccactagtattcacaagcttcgttcgcattccaccgaagtaccctgttgtacttaagggcatcgc
Oligo_P2_antiparallel_rv	ccgggcgatgcccttaagtacaacagggtacttcggtggaatgcgaacgaagcttgtgaatactagtg
5‘-HindIII-pC	attcaagcttATGAGTACTGCCCGCGATAAG
3‘-SpeI-pC	attcactagtTCATGTTTTATCATCCGGCCGAAG
5‘-HindIII-pN	attcaagcttATGACGAACGAGTCCCGTAAAC
3‘-SpeI-pN	attcactagtTTAAGAAAGGGCGACTTCCATG
5‘-HindIII-pO	attcaagcttATGGCAGATCCAGCAAACG
3‘-SpeI-pO	attcactagtCTACAGTTCCTCTTTCATGTCGC

CBD vectors

5‘-XbaI-pA	attctctagaATGGCGCAACCAGATTC
5‘-XbaI-pC	attctctagaATGAGTGTCACAGACAAACGCGACG
5‘-XbaI-pN	attctctagaATGACGAACGAGTCC
5‘-XbaI-pO	attctctagaATGGCAGATCCAGCAAACG
3‘-KpnI-pA	agttctggtaccTCAGGCCTCGGGTGC
3‘-BsrGI-pC	attctgtacaTCATGTTTTATCATCCGG
3‘-KpnI-pN	attcggtaccTTAAGAAAGGGCGACTTCCATG
3‘-KpnI-pO	attcggtaccCTACAGTTCCTCTTTCATGTC

underlined: recognition sequence for endonucleases

CAPITALS: annealing area

bold: altered nucleotide sequences to obtain the respective aa substitution in GvpF

Supplementary Table S2. Results of the ΔF+F_{mut} transformants and F_{mut}/A interaction studies.

Substitution	position	rf value F/A	GV width [nm]	GV length [nm]	GV shape*
GvpF wild type		18.0	165	376	wt
E03A	-	14.5	94	233	small GV
E03R	-	14.0	166	250	wt
E12A	-	14.7	200	460	wt
E12R	-	15.0	202	472	wt
E14A	-	15.8	127	506	small Ø + long
E14R	-	14.3	192	409	wt
D15A	-	12.9	213	619	cylinder-shaped
D15R	-	12.0	104	233	small GV
E17A	-	16.4	182	240	wt
E17R	-	14.7	181	434	wt
D19A	-	13.6	110	205	small GV
D19R	-	12.0	92	235	small GV
E21A	-	17.3	152	234	wt
E21R	-	15.8	172	301	wt
E27A	-	1.1	184	431	wt
E27R	-	2.1	192	451	wt
D45A	loop	13.0	186	383	wt
D45R	loop	12.6	184	389	wt
D48A	loop	15.8	175	384	wt
D48R	loop	15.3	183	450	wt
E50A	loop	14.8	208	425	wt
E50R	loop	15.7	163	300	wt
R51A	loop	15.1	188	399	wt
R51E	loop	10.1	159	316	wt
D53A	α1	13.7	154	328	wt
D53R	α1	12.7	205	618	cylinder-shaped
E54A	α1	14.3	180	657	cylinder-shaped
E54R	α1	14.4	162	412	wt
D55A	α1	15.3	188	433	wt
D55R	α1	12.0	152	413	wt
E57A	α1	16.5	185	831	cylinder-shaped
E57R	α1	16.6	226	995	cylinder-shaped
E65A	α1	15.7	109	654	small Ø + long
E65R	α1	15.3	162	384	wt
K68A	α1	15.7	173	448	wt
K68E	α1	15.2	223	813	cylinder-shaped
E70A	loop	14.5	240	845	cylinder-shaped
E70R	loop	14.1	162	449	wt
E71A	loop	13.8	179	567	cylinder-shaped
E71R	loop	16.6	-	-	Vac negative

Substitution	position	rf value	GV width [nm]	GV length [nm]	GV shape*
E72A	loop	15.1	-	-	Vac negative
E72R	loop	15.5	-	-	Vac negative
R73A	loop	12.3	222	687	cylinder-shaped
R73E	loop	5.9	171	313	
K85A	-	12.9	192	684	cylinder-shaped
K85E	-	10.8	154	277	wt
R88A	α2	12.4	156	181	wt
R88E	α2	13.6	182	396	wt
K91A	α2	10.3	177	227	wt
K91E	α2	12.8	178	361	wt
R95A	α2	15.2	153	316	wt
R95E	α2	15.3	186	350	wt
R98A	loop	17.7	193	274	wt
R98E	loop	17.8	136	225	wt
R102A	α3	15.5	163	256	wt
R102E	α3	16.3	163	335	wt
D123A	loop	18.3	173	403	wt
D123R	loop	16.6	173	451	wt
D124A	loop	16.8	163	435	wt
D124R	loop	16.4	172	436	wt
D154A	loop	11.2	145	324	wt
D154R	loop	14.0	151	360	wt
R155A	loop	19.6	164	301	wt
R155E	loop	18.4	162	332	wt
D184A	loop	13.3	160	393	wt
D184R	loop	15.2	154	316	wt
E185A	loop	17.1	178	324	wt
E185R	loop	11.2	144	280	wt
R213A	-	17.1	172	227	wt
R213E	-	15.8	-	-	Vac negative

*GV, gas vesicle; small, <110 nm ø and <233 nm in length (shaded in yellow); long, > 500 nm (shaded in blue)

Supplementary Table S3. Gvp interactions investigated by split GFP. The fluorescence was measured in LAU/mm^2 and the relative fluorescence was calculated.

transformant		LAU/mm^2	σ (LAU/mm^2)	Relative fluorecescence (rf)
control				
	WR340	17,066	1,687	0.00
interactions of GvpA, C, N and O				
$_N$A	A$_C$	14,172	659	0.00
	$_C$A	13,688	1594	0.00
A$_N$	A$_C$	13,687	1594	0.00
	$_C$A	15,417	2499	0.03
$_N$A	C$_C$	19,032	1,836	0.19
	$_C$C	10,936	1,524	0.00
A$_N$	C$_C$	13,146	733	0.00
	$_C$C	12,218	1,031	0.00
$_C$A	C$_N$	11,560	2,708	0.00
	$_N$C	11,444	1,041	0.00
A$_C$	C$_N$	17,049	821	0.07
	$_N$C	28,078	2,703	0.76
$_N$A	N$_C$	15,575	846	0.01
	$_C$N	13,872	549	0.00
A$_N$	N$_C$	13,509	1,684	0.01
	$_C$N	13,861	623	0.00
$_C$A	N$_N$	12,681	750	0.00
	$_N$N	12,915	2,444	0.00
A$_C$	N$_N$	14,101	1,169	0.00
	$_N$N	43,322	9,522	1.71
$_N$A	O$_C$	21,647	570	0.36
	$_C$O	27,193	607	0.70
A$_N$	O$_C$	16,640	2,860	0.10
	$_C$O	25,875	964	0.62
$_C$A	O$_N$	26,058	1,358	0.63
	$_N$O	12,874	961	0.00
A$_C$	O$_N$	15,987	1,524	0.03
	$_N$O	23,269	758	0.46
$_N$C	C$_C$	234,565	6,484	13.81
	$_C$C	23,228	793	0.47
C$_N$	C$_C$	174,549	3,767	10.02
	$_C$C	20,355	1,082	0.29
$_N$C	N$_C$	115,598	2,729	6.24
	$_C$N	12,779	1,007	0.00
C$_N$	N$_C$	31,279	4,215	0.96
	$_C$N	13,526	791	0.00
$_C$C	N$_N$	43,406	2,939	1.72
	$_N$N	17,727	911	0.11
C$_C$	N$_N$	12,251	820	0.00
	$_N$N	12,751	331	0.00
$_N$C	O$_C$	89,422	11,263	4.60
	$_C$O	13,214	825	0.00
C$_N$	O$_C$	32,861	1,573	1.06
	$_C$O	14,828	916	0.00
$_C$C	O$_N$	34,740	609	1.18
	$_N$O	20,469	1,871	0.28
C$_C$	O$_N$	13,484	1,505	0.00
	$_N$O	14,576	461	0.00
$_N$N	N$_C$	306,481	49,736	18.19
	$_C$N	19,170	636	0.20
N$_N$	N$_C$	62,619	9,783	2.92
	$_C$N	19,359	690	0.21
$_N$N	O$_C$	215,223	18,985	12.48
	$_C$O	23,128	2,269	0.45
N$_N$	O$_C$	55,636	5,625	2.48
	$_C$O	21,054	2,365	0.32
$_C$N	O$_N$	21,794	3,803	0.36
	$_N$O	18,518	2,107	0.16
N$_C$	O$_N$	89,310	10,267	4.59
	$_N$O	134,117	13,785	7.40

transformant		LAU/mm^2	σ (LAU/mm^2)	Relative fluorecescence (rf)
$_N$O	O$_C$	189,956	7,265	10.89
	$_C$O	35,669	1,940	1.23
O$_N$	O$_C$	131,321	4,530	7.22
	$_C$O	34,747	2,528	1.18
interactions of GvpA fragments with GvpA, C, N and O				
$_N$A	A1-22$_C$	15,826	1,837	0.04
	$_C$A1-22	12,810	748	0.00
A$_N$	A1-22$_C$	13,962	526	0.00
	$_C$A1-22	11,711	431	0.00
$_C$A	A1-22$_N$	11,340	381	0.00
	$_N$A1-22	11,912	1,533	0.00
A$_C$	A1-22$_N$	12,006	1,108	0.00
	$_N$A1-22	20,026	1,903	0.25
$_N$A	A1-34$_C$	16,171	736	0.03
	$_C$A1-34	14,529	768	0.00
A$_N$	A1-34$_C$	13,951	778	0.00
	$_C$A1-34	14,397	436	0.00
$_C$A	A1-34$_N$	15,088	748	0.01
	$_N$A1-34	12,626	759	0.00
A$_C$	A1-34$_N$	15,751	780	0.02
	$_N$A1-34	22,535	1,427	0.41
$_N$A	A1-43$_C$	16,090	1,105	0.04
	$_C$A1-43	14,284	1,061	0.00
A$_N$	A1-43$_C$	13,580	976	0.00
	$_C$A1-43	15,288	818	0.00
$_C$A	A1-43$_N$	15,391	841	0.01
	$_N$A1-43	12,408	1,440	0.00
A$_C$	A1-43$_N$	15,841	1,695	0.04
	$_N$A1-43	17,966	1,930	0.13
$_N$A	A20-47$_C$	16,203	2,180	0.00
	$_C$A20-47	21,149	1,226	0.00
A$_N$	A20-47$_C$	21,927	2,149	0.05
	$_C$A20-47	22,195	794	0.02
$_C$A	A20-47$_N$	19,927	3,554	0.03
	$_N$A20-47	18,256	3,569	0.01
A$_C$	A20-47$_N$	22,685	818	0.03
	$_N$A20-47	21,703	4,770	0.10
$_N$A	A44-76$_C$	11,376	3,078	0.00
	$_C$A44-76	13,190	1,960	0.00
A$_N$	A44-76$_C$	13,604	706	0.00
	$_C$A44-76	11,301	3,089	0.00
$_C$A	A44-76$_N$	12,304	1,208	0.00
	$_N$A44-76	12,496	1,680	0.00
A$_C$	A44-76$_N$	13,359	752	0.00
	$_N$A44-76	14,333	2,252	0.02
$_N$C	A1-22$_C$	27,565	2,234	0.73
	$_C$A1-22	13,417	433	0.00
C$_N$	A1-22$_C$	16,704	432	0.05
	$_C$A1-22	11,700	2,162	0.00
$_C$C	A1-22$_N$	66,800	3,325	3.18
	$_N$A1-22	17,868	1,379	0.12
C$_C$	A1-22$_N$	14,736	1,772	0.02
	$_N$A1-22	13,244	1,669	0.00
$_N$C	A1-34$_C$	28,687	2,047	0.80
	$_C$A1-34	22,223	1,487	0.39
C$_N$	A1-34$_C$	20,622	1,371	0.29
	$_C$A1-34	19,450	694	0.22
$_C$C	A1-34$_N$	19,389	2,403	0.80
	$_N$A1-34	17,489	1,983	0.11
C$_C$	A1-34$_N$	23,111	2,404	0.45
	$_N$A1-34	31,914	3,089	1.00

transformant		LAU/mm^2	σ (LAU/mm^2)	Relative fluorecescence (rf)
$_N$C	A1-43$_C$	46,501	3,529	1.91
	$_C$A1-43	37,637	6,435	1.36
C$_N$	A1-43$_C$	32,630	1,393	1.04
	$_C$A1-43	38,048	12,674	1.38
$_C$C	A1-43$_N$	26,045	2,228	0.63
	$_N$A1-43	31,530	1,827	0.92
C$_C$	A1-43$_N$	27,645	1,963	0.57
	$_N$A1-43	64,102	3,656	3.01
$_N$C	A20-47$_C$	19,229	681	0.00
	$_C$A20-47	19,675	1,298	0.03
C$_N$	A20-47$_C$	19,229	681	0.00
	$_C$A20-47	17,015	585	0.00
$_C$C	A20-47$_N$	15,580	420	0.00
	$_N$A20-47	17,433	1,011	0.00
C$_C$	A20-47$_N$	20,671	4,369	0.09
	$_N$A20-47	19,735	739	0.02
$_N$C	A44-76$_C$	18,984	3,699	0.21
	$_C$A44-76	23,609	24,847	0.66
C$_N$	A44-76$_C$	14,589	1110	0.00
	$_C$A44-76	13,724	1043	0.00
$_C$C	A44-76$_N$	26,037	3023	0.63
	$_N$A44-76	21,809	1040	0.37
C$_C$	A44-76$_N$	12,996	1337	0.00
	$_N$A44-76	13,821	625	0.00
$_N$N	A1-22$_C$	42,909	5,851	1.69
	$_C$A1-22	24,427	2,508	0.53
N$_N$	A1-22$_C$	24,377	2,165	0.53
	$_C$A1-22	19,353	2,254	0.21
$_C$N	A1-22$_N$	54,368	7,857	2.40
	$_N$A1-22	28,056	2,319	0.76
N$_C$	A1-22$_N$	22,259	1,137	0.39
	$_N$A1-22	20,883	3,354	0.31
$_N$N	A1-34$_C$	25408	5,693	0.59
	$_C$A1-34	17,265	1,619	0.10
N$_N$	A1-34$_C$	14,588	2,529	0.04
	$_C$A1-34	18,040	1,429	0.13
$_C$N	A1-34$_N$	17,082	1,405	0.08
	$_N$A1-34	13,327	1,319	0.00
N$_C$	A1-34$_N$	16,676	2,485	0.10
	$_N$A1-34	18,964	1,693	0.19
$_N$N	A1-43$_C$	45,116	2,232	1.82
	$_C$A1-43	26,932	8,770	0.69
N$_N$	A1-43$_C$	39,904	4,756	1.50
	$_C$A1-43	26,331	1,409	0.65
$_C$N	A1-43$_N$	22,462	2,279	0.41
	$_N$A1-43	22,761	3,078	0.43
N$_C$	A1-43$_N$	25,932	2,576	0.62
	$_N$A1-43	42,928	8,540	1.69
$_N$N	A20-47$_C$	17,156	1,202	0.00
	$_C$A20-47	17,472	1,143	0.00
N$_N$	A20-47$_C$	18,720	849	0.00
	$_C$A20-47	19,287	916	0.01
$_C$N	A20-47$_N$	15,427	1,431	0.00
	$_N$A20-47	16,072	340	0.00
N$_C$	A20-47$_N$	18,575	883	0.00
	$_N$A20-47	19,160	1,394	0.02
$_N$N	A44-76$_C$	444,722	10,140	1.80
	$_C$A44-76	13,047	537	0.00
N$_N$	A44-76$_C$	18,136	1,700	0.14
	$_C$A44-76	12,303	1,727	0.00
$_C$N	A44-76$_N$	17,023	992	0.07
	$_N$A44-76	20,433	2,469	0.28
N$_C$	A44-76$_N$	12,528	1,371	0.00
	$_N$A44-76	12,523	1,082	0.00

transformant		LAU/mm^2	σ (LAU/mm^2)	Relative fluorecescence (rf)
$_N$O	A1-22$_C$	15,649	498	0.00
	$_C$A1-22	15,363	941	0.00
O$_N$	A1-22$_C$	13,788	299	0.00
	$_C$A1-22	15,123	714	0.00
$_C$O	A1-22$_N$	15,195	1,181	0.01
	$_N$A1-22	16,286	1,251	0.05
O$_C$	A1-22$_N$	34,687	1,911	1.17
	$_N$A1-22	64,398	12,075	3.03
$_N$O	A1-34$_C$	18,171	2,148	0.14
	$_C$A1-34	13,056	730	0.00
O$_N$	A1-34$_C$	14,160	2,448	0.03
	$_C$A1-34	15,691	1,636	0.03
$_C$O	A1-34$_N$	13,730	3,477	0.04
	$_N$A1-34	11,583	747	0.00
O$_C$	A1-34$_N$	21,103	1,909	0.32
	$_N$A1-34	33,205	4,285	1.08
$_N$O	A1-43$_C$	12,699	1,156	0.00
	$_C$A1-43	11,649	1,340	0.00
O$_N$	A1-43$_C$	11,279	1,653	0.00
	$_C$A1-43	11,142	1,417	0.00
$_C$O	A1-43$_N$	12,336	2,850	0.01
	$_N$A1-43	11,515	732	0.00
O$_C$	A1-43$_N$	14,813	1,706	0.02
	$_N$A1-43	22,813	1,869	0.43
$_N$O	A20-47$_C$	16,511	852	0.00
	$_C$A20-47	15,077	1,711	0.00
O$_N$	A20-47$_C$	19,434	1,336	0.02
	$_C$A20-47	18,386	1,209	0.00
$_C$O	A20-47$_N$	18,007	880	0.00
	$_N$A20-47	18,408	643	0.00
O$_C$	A20-47$_N$	18,738	2,177	0.03
	$_N$A20-47	19,873	1,930	0.05
$_N$O	A44-76$_C$	25,797	559	0.62
	$_C$A44-76	26,852	1,053	0.68
O$_N$	A44-76$_C$	27,617	1,035	0.73
	$_C$A44-76	26,055	747	0.63
$_C$O	A44-76$_N$	23,271	1,197	0.46
	$_N$A44-76	26,876	2,074	0.68
O$_C$	A44-76$_N$	32,550	1,459	1.04
	$_N$A44-76	30,984	1,330	0.94
Interactions between GvpA fragements				
$_N$A1-22	A1-22$_C$	21,993	1,421	0.38
	$_C$A1-22	15,950	753	0.02
A1-22$_N$	A1-22$_C$	16,726	1,066	0.06
	$_C$A1-22	14,837	753	0.00
$_N$A1-22	A1-34$_C$	23,576	478	0.48
	$_C$A1-34	14,961	775	0.00
A1-22$_N$	A1-34$_C$	14,314	899	0.00
	$_C$A1-34	14,519	495	0.00
$_C$A1-22	A1-34$_N$	14,361	2,068	0.02
	$_N$A1-34	12,537	1,788	0.00
A1-22$_C$	A1-34$_N$	15,394	1,788	0.00
	$_N$A1-34	20,398	776	0.28
$_N$A1-22	A1-43$_C$	14,411	558	0.00
	$_C$A1-43	11,671	1,386	0.00
A1-22$_N$	A1-43$_C$	12,026	1,630	0.00
	$_C$A1-43	13,205	624	0.00
$_C$A1-22	A1-43$_N$	14,450	1,481	0.00
	$_N$A1-43	11,880	2,088	0.00
A1-22$_C$	A1-43$_N$	12,202	1,026	0.00
	$_N$A1-43	16,512	702	0.04

transformant		LAU/mm^2	σ (LAU/mm^2)	Relative fluorecescence (rf)
$_N$A1-22	A20-47$_C$	22,577	4,535	0.01
	$_C$A20-47	20,726	1,371	0.00
A1-22$_N$	A20-47$_C$	22,433	4,361	0.01
	$_C$A20-47	23,592	3,915	0.02
$_C$A1-22	A20-47$_N$	21,664	1,959	0.00
	$_N$A20-47	16,571	1,187	0.00
A1-22$_C$	A20-47$_N$	21,521	2,866	0.00
	$_N$A20-47	16,929	382	0.00
$_N$A1-22	A44-76$_C$	13,629	918	0.00
	$_C$A44-76	10,859	487	0.00
A1-22$_N$	A44-76$_C$	14,530	3,837	0.07
	$_C$A44-76	10,575	943	0.00
$_C$A1-22	A44-76$_N$	18,168	2,166	0.15
	$_N$A44-76	13,629	320	0.00
A1-22$_C$	A44-76$_N$	11,038	1,202	0.00
	$_N$A44-76	9757	1,121	0.00
$_N$A1-34	A1-34$_C$	19,940	1,236	0.25
	$_C$A1-34	15,892	1,302	0.04
A1-34$_N$	A1-34$_C$	13,930	1,015	0.00
	$_C$A1-34	16,870	354	0.06
$_N$A1-34	A1-43$_C$	15,726	1,841	0.05
	$_C$A1-43	13,537	2,415	0.00
A1-34$_N$	A1-43$_C$	13,602	208	0.00
	$_C$A1-43	14,361	1,618	0.01
$_C$A1-34	A1-43$_N$	12,388	479	0.00
	$_N$A1-43	12,338	1,536	0.00
A1-34$_C$	A1-43$_N$	13,197	553	0.00
	$_N$A1-43	14,540	1,917	0.01
$_N$A1-34	A20-47$_C$	19,048	2,443	0.00
	$_C$A20-47	18,391	2,423	0.00
A1-34$_N$	A20-47$_C$	19,651	818	0.00
	$_C$A20-47	18,996	1,393	0.00
$_C$A1-34	A20-47$_N$	18,982	2,025	0.00
	$_N$A20-47	17,154	878	0.00
A1-34$_C$	A20-47$_N$	18,143	2,382	0.00
	$_N$A20-47	15,917	533	0.00
$_N$A1-34	A44-76$_C$	12,016	1,478	0.00
	$_C$A44-76	13,097	1,382	0.00
A1-34$_N$	A44-76$_C$	12,959	362	0.00
	$_C$A44-76	11,171	1,181	0.00
$_C$A1-34	A44-76$_N$	11,575	1,049	0.00
	$_N$A44-76	14,422	1,578	0.02
A1-34$_C$	A44-76$_N$	12,091	1,706	0.00
	$_N$A44-76	10,569	1,622	0.00
$_N$A1-43	A1-43$_C$	17,508	828	0.10
	$_C$A1-43	14,560	594	0.00
A1-43$_N$	A1-43$_C$	15,445	983	0.01
	$_C$A1-43	14,698	300	0.00
$_N$A1-43	A20-47$_C$	19,048	2,443	0.00
	$_C$A20-47	18,391	2,423	0.00
A1-43$_N$	A20-47$_C$	19,651	818	0.00
	$_C$A20-47	18,996	1,393	0.00
$_C$A1-43	A20-47$_N$	18,982	2,025	0.00
	$_N$A20-47	17,154	878	0.00
A1-43$_C$	A20-47$_N$	18,143	2,382	0.00
	$_N$A20-47	15,917	533	0.00
$_N$A1-43	A44-76$_C$	13,454	827	0.00
	$_C$A44-76	10,883	1,843	0.00
A1-43$_N$	A44-76$_C$	13,857	1,618	0.00
	$_C$A44-76	14,367	859	0.00
$_C$A1-43	A44-76$_N$	16,299	2,173	0.07
	$_N$A44-76	11,438	1,641	0.00
A1-43$_C$	A44-76$_N$	13,654	959	0.00
	$_N$A44-76	15,455	2,659	0.07

transformant		LAU/mm^2	σ (LAU/mm^2)	Relative fluorecescence (rf)
$_N$A20-47	A20-47$_C$	23,518	438	0.07
	$_C$A20-47	20,238	994	0.00
A20-47$_N$	A20-47$_C$	25,163	1,192	0.14
	$_C$A20-47	23,573	916	0.07
$_N$A20-47	A44-76$_C$	17,282	1,242	0.00
	$_C$A44-76	15,676	448	0.00
A20-47$_N$	A44-76$_C$	19,773	796	0.00
	$_C$A44-76	18,613	975	0.00
$_C$A20-47	A44-76$_N$	17,872	1,728	0.00
	$_N$A44-76	18,250	1,272	0.00
A20-47$_C$	A44-76$_N$	19,786	2,469	0.01
	$_N$A44-76	20,558	1,282	0.00
Interactions with GvpC fragements				
$_N$C_N	A$_C$	44,007	3,379	2.03
	$_C$A	16,535	1,058	0.14
C_N$_N$	A$_C$	22,202	2,650	0.53
	$_C$A	16,911	1,808	0.17
$_C$C_N	A$_N$	24,181	1,736	0.67
	$_N$A	23,550	3,354	0.62
C_N$_C$	A$_N$	18,577	848	0.28
	$_N$A	16,323	3,286	0.15
$_N$C_N	C$_C$	183,013	34,749	11.61
	$_C$C	23,134	1,172	0.59
C_N$_N$	C$_C$	63,862	11,231	3.40
	$_C$C	23,111	1,689	0.59
$_C$C_N	C$_N$	267,874	26,972	17.46
	$_N$C	63,188	5,175	3.35
C_N$_C$	C$_N$	24,350	767	0.68
	$_N$C	20,897	1,699	0.44
$_N$C_N	N$_C$	81,680	5,780	4.63
	$_C$N	20,408	567	0.41
C_N$_N$	N$_C$	34,978	3,528	1.41
	$_C$N	17,781	2,559	0.23
$_C$C_N	N$_N$	90,244	3,217	5.22
	$_N$N	25,106	1,39	0.73
C_N$_C$	N$_N$	14,357	1,650	0.04
	$_N$N	16,580	742	0.14
$_N$C_N	O$_C$	80,164	6,053	4.52
	$_C$O	18,836	2,570	0.30
C_N$_N$	O$_C$	45,475	3,480	2.13
	$_C$O	15,368	1,388	0.07
$_C$C_N	O$_N$	48,207	4,290	2.32
	$_N$O	29,787	5,913	1.05
C_N$_C$	O$_N$	19,116	4,042	0.32
	$_N$O	20,318	5,483	0.41
$_N$C_N	A1-22$_C$	27,447	1,156	0.39
	$_C$A1-22	14,565	1,879	0.00
C_N$_N$	A1-22$_C$	22,720	1,851	0.15
	$_C$A1-22	14,156	1,315	0.00
$_C$C_N	A1-22$_N$	90,434	1,875	3.57
	$_N$A1-22	19,853	3,075	0.07
C_N$_C$	A1-22$_N$	17,311	1,001	0.00
	$_N$A1-22	14,022	2,611	0.00
$_N$C_N	A1-34$_C$	17,900	1,194	0.02
	$_C$A1-34	15,487	1,963	0.00
C_N$_N$	A1-34$_C$	16,409	1,684	0.01
	$_C$A1-34	16,299	1,194	0.00
$_C$C_N	A1-34$_N$	21,386	2,240	0.16
	$_N$A1-34	16,609	1,884	0.00
C_N$_C$	A1-34$_N$	12,428	719	0.00
	$_N$A1-34	12,961	559	0.00

transformant		LAU/mm²	σ (LAU/mm²)	Relative fluorecescence (rf)
$_N$C_N	A1-43$_C$	14,106	612	0.00
	$_C$A1-43	14,289	1,559	0.00
C_N$_N$	A1-43$_C$	13,731	2,321	0.00
	$_C$A1-43	14,914	737	0.00
$_C$C_N	A1-43$_N$	18,466	1,686	0.12
	$_N$A1-43	14,003	1,205	0.00
C_N$_C$	A1-43$_N$	12,200	523	0.00
	$_N$A1-43	13,340	1,771	0.00
$_N$C_N	A20-47$_C$	12,454	584	0.00
	$_C$A20-47	12,841	517	0.00
C_N$_N$	A20-47$_C$	12,733	1,169	0.00
	$_C$A20-47	13,042	1,215	0.00
$_C$C_N	A20-47$_N$	11,847	739	0.00
	$_N$A20-47	12,779	478	0.00
C_N$_C$	A20-47$_N$	12,334	792	0.00
	$_N$A20-47	11,984	2,021	0.00
$_N$C_N	A44-76$_C$	32,604	2,780	1.15
	$_C$A44-76	12,720	1,074	0.00
C_N$_N$	A44-76$_C$	25,524	2,395	0.68
	$_C$A44-76	12,856	1,106	0.00
$_C$C_N	A44-76$_N$	31,792	4,220	1.09
	$_N$A44-76	33,808	1,722	1.23
C_N$_C$	A44-76$_N$	13,085	1,763	0.01
	$_N$A44-76	11,156	512	0.00
$_N$C_N	F$_C$	56,651	11,317	4.74
	$_C$F	9,337	611	0.01
C_N$_N$	F$_C$	42,300	1,571	3.29
	$_C$F	8,694	789	0.00
$_C$C_N	F$_N$	30,075	1,055	2.05
	$_N$F	61,773	7,350	5.26
C_N$_C$	F$_N$	6,948	1,433	0.03
	$_N$F	7,635	833	0.01
$_N$C_N	H$_C$	54,982	1,796	5.63
	$_C$H	7,796	1,528	0.06
C_N$_N$	H$_C$	49,483	3,867	4.96
	$_C$H	6,649	821	0.00
$_C$C_N	H$_N$	26,532	2,859	2.20
	$_N$H	34,482	5,207	3.16
C_N$_C$	H$_N$	4,786	190	0.00
	$_N$H	6,363	1,557	0.02
$_N$C_N	I$_C$	11,815	2,364	0.00
	$_C$I	14,367	1,054	0.01
C_N$_N$	I$_C$	12,527	1,415	0.00
	$_C$I	14,706	468	0.00
$_C$C_N	I$_N$	87,463	6,996	4.55
	$_N$I	48,659	2,821	2.09
C_N$_C$	I$_N$	13,219	1,948	0.00
	$_N$I	15,864	1,445	0.04
$_N$C_N	L$_C$	58,019	11,029	2.68
	$_C$L	14,784	660	0.00
C_N$_N$	L$_C$	50,600	7,394	2.21
	$_C$L	12,927	828	0.00
$_C$C_N	L$_N$	143,546	17,646	9.07
	$_N$L	23,183	2,164	0.63
C_N$_C$	L$_N$	13,129	524	0.00
	$_N$L	12,955	538	0.00
$_N$C_N	C_N$_C$	77,698	1,975	4.35
	$_C$C_N	20,655	1,910	0.42
C_N$_N$	C_N$_C$	33,157	12,837	1.29
	$_C$C_N	22,341	1,192	0.54

transformant		LAU/mm²	σ (LAU/mm²)	Relative fluorecescence (rf)
$_N$C_C	A$_C$	70,679	7,701	2.46
	$_C$A	28,831	3,028	0.41
C_C$_N$	A$_C$	32,942	2,402	0.61
	$_C$A	34,794	3,948	0.70
$_C$C_C	A$_N$	38,940	15,534	1.03
	$_N$A	29,231	1,266	0.43
C_C$_C$	A$_N$	22,427	4,265	0.15
	$_N$A	29,205	2,476	0.43
$_N$C_C	C$_C$	397,952	30,570	18.47
	$_C$C	24,437	1,120	0.20
C_C$_N$	C$_C$	171,764	16,119	7.40
	$_C$C	29,926	3,151	0.46
$_C$C_C	C$_N$	118,035	9,876	4.78
	$_N$C	54,329	1,270	1.66
C_C$_C$	C$_N$	21,628	1,365	0.07
	$_N$C	21,614	1,772	0.07
$_N$C_C	N$_C$	228,695	46,126	10.19
	$_C$N	28,644	6,686	0.40
C_C$_N$	N$_C$	94,792	16,078	3.64
	$_C$N	29,993	2,257	0.47
$_C$C_C	N$_N$	93,085	5,475	3.55
	$_N$N	33,054	4,274	0.62
C_C$_C$	N$_N$	23,951	2,898	0.18
	$_N$N	18,755	1,156	0.00
$_N$C_C	O$_C$	292,070	9,921	15.98
	$_C$O	24,511	1,084	0.42
C_C$_N$	O$_C$	146,664	18,302	7.53
	$_C$O	20,020	2,685	0.18
$_C$C_C	O$_N$	49,710	4,782	1.89
	$_N$O	30,462	2,759	0.77
C_C$_C$	O$_N$	17,182	1,991	0.04
	$_N$O	17,609	1,320	0.05
$_N$C_C	A1-22$_C$	26,085	2,917	0.32
	$_C$A1-22	19,332	1,832	0.03
C_C$_N$	A1-22$_C$	22,833	516	0.15
	$_C$A1-22	19,103	809	0.00
$_C$C_C	A1-22$_N$	42,333	324	1.14
	$_N$A1-22	27,951	4,588	0.41
C_C$_C$	A1-22$_N$	20,331	1,763	0.06
	$_N$A1-22	18,447	527	0.00
$_N$C_C	A1-34$_C$	50,613	4,041	2.07
	$_C$A1-34	15,273	904	0.00
C_C$_N$	A1-34$_C$	29,512	3,598	0.79
	$_C$A1-34	14,346	1,322	0.00
$_C$C_C	A1-34$_N$	54,105	912	2.64
	$_N$A1-34	21,230	673	0.43
C_C$_C$	A1-34$_N$	11,602	915	0.00
	$_N$A1-34	13,160	1,052	0.00
$_N$C_C	A1-43$_C$	32,318	1,068	1.17
	$_C$A1-43	15,575	5,691	0.15
C_C$_N$	A1-43$_C$	20,413	491	0.37
	$_C$A1-43	11,632	745	0.00
$_C$C_C	A1-43$_N$	29,105	14,431	1.65
	$_N$A1-43	12,026	1,685	0.00
C_C$_C$	A1-43$_N$	20,108	17,593	0.50
	$_N$A1-43	23,128	12,338	0.00
$_N$C_C	A20-47$_C$	19,203	1,422	0.02
	$_C$A20-47	19,047	2,265	0.04
C_C$_N$	A20-47$_C$	18,268	1,488	0.01
	$_C$A20-47	17,381	671	0.00
$_C$C_C	A20-47$_N$	23,554	712	0.19
	$_N$A20-47	20,191	1,849	0.06
C_C$_C$	A20-47$_N$	17,346	655	0.00
	$_N$A20-47	19,237	705	0.01

transformant		LAU/mm^2	σ (LAU/mm^2)	Relative fluorecescence (rf)
$_N$C_C	A44-76$_C$	23,134	540	0.00
	$_C$A44-76	18,378	1,381	0.00
C_C$_N$	A44-76$_C$	20,857	792	0.00
	$_C$A44-76	19,320	1,249	0.00
$_C$C_C	A44-76$_N$	30,463	934	0.15
	$_N$A44-76	33,537	1,431	0.27
C_C$_C$	A44-76$_N$	19,737	524	0.00
	$_N$A44-76	17,507	2,358	0.00
$_N$C_C	F$_C$	278,322	11,509	18.52
	$_C$F	19,165	3,139	0.34
C_C$_N$	F$_C$	91,787	7,826	5.44
	$_C$F	13,140	2,084	0.04
$_C$C_C	F$_N$	138,623	2,582	8.72
	$_N$F	102,558	6,438	6.19
C_C$_C$	F$_N$	12,735	805	0.00
	$_N$F	11,832	1,003	0.00
$_N$C_C	H$_C$	536,186	147,706	42.59
	$_C$H	19,880	1,878	0.62
C_C$_N$	H$_C$	254,327	42,437	19.68
	$_C$H	16,504	1,639	0.34
$_C$C_C	H$_N$	141,494	32,538	10.50
	$_N$H	115,989	1,729	8.43
C_C$_C$	H$_N$	16,506	1,210	0.34
	$_N$H	15,324	2,626	0.25
$_N$C_C	I$_C$	13,639	1,406	0.00
	$_C$I	22,438	807	0.19
C_C$_N$	I$_C$	15,413	1,115	0.00
	$_C$I	17,274	1,407	0.11
$_C$C_C	I$_N$	297,812	16,750	18.13
	$_N$I	198,161	4,965	11.73
C_C$_C$	I$_N$	20,790	402	0.34
	$_N$I	19,418	327	0.25
$_N$C_C	L$_C$	499,920	87,077	31.11
	$_C$L	22,109	497	0.42
C_C$_N$	L$_C$	354,947	22,555	21.80
	$_C$L	19,760	1,270	0.27
$_C$C_C	L$_N$	449,455	28,319	27.87
	$_N$L	78,147	7,607	4.02
C_C$_C$	L$_N$	21,352	1,513	0.37
	$_N$L	13,610	599	0.00
$_N$C_C	C_N$_C$	191,027	8,570	10.10
	$_C$C_N	17,501	840	0.03
C_C$_N$	C_N$_C$	91,964	13,522	4.35
	$_C$C_N	18,659	1,039	0.09
$_C$C_C	C_N$_N$	56,865	3,358	2.31
	$_N$C_N	32,266	1,273	0.88
C_C$_C$	C_N$_N$	21,760	3,276	0.26
	$_N$C_N	20,276	1,959	0.18
$_N$C_C	C_C$_C$	376,499	66,338	20.73
	$_C$C_C	27,597	1,590	0.59
C_C$_N$	C_C$_C$	147,748	39,155	7.53
	$_C$C_C	25,560	4,208	0.47
Interactions of GvpC with accessory proteins GvpF - GvpM				
$_N$C	F$_C$	110,199	8,089	3.62
	$_C$F	16,912	699	0.00
C$_N$	F$_C$	79,813	39,818	2.34
	$_C$F	18,401	614	0.00
$_C$C	F$_N$	146,230	2,273	3.91
	$_N$F	298,162	12,172	9.00
C$_C$	F$_N$	23,084	2,614	0.00
	$_N$F	21,109	1,309	0.00

transformant		LAU/mm^2	σ (LAU/mm^2)	Relative fluorecescence (rf)
$_N$C	G$_C$	126,397	13,111	3.91
	$_C$G	20,143	441	0.00
C$_N$	G$_C$	42,417	2,633	0.65
	$_C$G	19,403	714	0.00
$_C$C	G$_N$	44,828	2,171	0.74
	$_N$G	57,836	9,455	1.25
C$_C$	G$_N$	13,518	878	0.00
	$_N$G	15,675	457	0.00
$_N$C	H$_C$	218,528	18,363	7.49
	$_C$H	19,241	2,248	0.00
C$_N$	H$_C$	114,710	29,539	3.23
	$_C$H	20,887	1,836	0.00
$_C$C	H$_N$	119,856	10,453	3.42
	$_N$H	202,966	42,276	6.49
C$_C$	H$_N$	19,674	734	0.00
	$_N$H	20,041	863	0.00
$_N$C	I$_C$	16,849	1,114	0.00
	$_C$I	19,375	1,695	0.00
C$_N$	I$_C$	19,463	382	0.00
	$_C$I	17,619	1,653	0.00
$_C$C	I$_N$	366,811	25,862	12.53
	$_N$I	190,749	14,747	6.03
C$_C$	I$_N$	23,731	1,375	0.00
	$_N$I	15,545	1,427	0.00
$_N$C	J$_C$	72,216	3,796	2.97
	$_C$J	17,846	394	0.00
C$_N$	J$_C$	40,343	2,571	1.22
	$_C$J	16,016	1,335	0.00
$_C$C	J$_N$	33,138	3,167	0.82
	$_N$J	33,783	1,358	0.86
C$_C$	J$_N$	13,206	1,378	0.00
	$_N$J	17,207	1,446	0.01
$_N$C	K$_C$	115,032	12,222	4.98
	$_C$K	23,021	2,654	0.20
C$_N$	K$_C$	66,384	3,239	2.45
	$_C$K	20,266	928	0.05
$_C$C	K$_N$	139,009	6,613	6.23
	$_N$K	51,548	7,761	1.68
C$_C$	K$_N$	18,182	517	0.00
	$_N$K	16,916	964	0.00
$_N$C	L$_C$	316,769	29,455	15.,47
	$_C$L	20,509	857	0.07
C$_N$	L$_C$	162,383	6364	7.44
	$_C$L	16,796	917	0.00
$_C$C	L$_N$	570,253	75,955	28.65
	$_N$L	113,629	15,816	4.13
C$_C$	L$_N$	31,035	875	0.40
	$_N$L	20,813	1,301	0.00
$_N$C	M$_C$	39,705	1,744	0.69
	$_C$M	15,130	663	0.00
C$_N$	M$_C$	18,951	1,819	0.00
	$_C$M	14,570	1,575	0.00
$_C$C	M$_N$	24,930	1,727	0.07
	$_N$M	18,645	1,431	0.00
C$_C$	M$_N$	11,678	313	0.00
	$_N$M	14,219	478	0.00
Interactions of GvpN with accessory proteins GvpF - GvpM				
$_N$N	F$_C$	93,109	12,516	2.52
	$_C$F	17,682	897	0.00
N$_N$	F$_C$	27,282	1,377	0.04
	$_C$F	20,293	1,127	0.00
$_C$N	F$_N$	72,603	11,097	1.75
	$_N$F	93,675	19,297	2.55
N$_C$	F$_N$	21,504	1,348	0.00
	$_N$F	24,606	1,986	0.01

transformant		LAU/mm²	σ (LAU/mm²)	Relative fluorecescence (rf)
$_{N}N$	G_C	95,657	8,569	2.62
	$_{C}G$	18,826	1,296	0.00
N_N	G_C	21,624	811	0.00
	$_{C}G$	18,517	889	0.00
$_{C}N$	G_N	20,099	2,321	0.00
	$_{N}G$	24,472	2,097	0.01
N_C	G_N	15,109	1,056	0.00
	$_{N}G$	18,461	998	0.00
$_{N}N$	H_C	114,166	8,098	3.41
	$_{C}H$	19,343	2,492	0.00
N_N	H_C	29,447	1,945	0.14
	$_{C}H$	17,133	2,768	0.00
$_{C}N$	H_N	32,801	1,638	0.27
	$_{N}H$	36,736	3,059	0.42
N_C	H_N	25,145	1,012	0.00
	$_{N}H$	23,365	2,897	0.01
$_{N}N$	I_C	24,078	777	0.00
	$_{C}I$	20,347	1,912	0.00
N_N	I_C	22,957	2,223	0.01
	$_{C}I$	18,659	1,274	0.00
$_{C}N$	I_N	140,825	8,163	4.44
	$_{N}I$	79,062	6,809	2.05
N_C	I_N	28,273	2,443	0.10
	$_{N}I$	20,191	1,573	0.00
$_{N}N$	J_C	57,941	8,661	1.55
	$_{C}J$	14,474	908	0.00
N_N	J_C	19,099	3,812	0.01
	$_{C}J$	13,128	1,441	0.00
$_{C}N$	J_N	18,030	749	0.00
	$_{N}J$	18,305	746	0.00
N_C	J_N	13,694	990	0.00
	$_{N}J$	12,970	684	0.00
$_{N}N$	K_C	59,428	6,143	1.35
	$_{C}K$	15,725	1,164	0.00
N_N	K_C	24,663	1,848	0.02
	$_{C}K$	15,266	1,046	0.00
$_{C}N$	K_N	47,568	768	0.88
	$_{N}K$	23,069	1,989	0.02
N_C	K_N	16,035	822	0.00
	$_{N}K$	14,444	421	0.00
$_{N}N$	L_C	195,931	61,705	7.06
	$_{C}L$	16,981	1,434	0.00
N_N	L_C	57,322	4,706	1.36
	$_{C}L$	15,315	748	0.00
$_{C}N$	L_N	435,927	8,924	16.92
	$_{N}L$	80,543	28,605	2.31
N_C	L_N	27,857	1,020	0.15
	$_{N}L$	16,062	1,867	0.00
$_{N}N$	M_C	77,137	19,198	2.17
	$_{C}M$	14,011	824	0.00
N_N	M_C	14,042	590	0.00
	$_{C}M$	13,662	1,381	0.00
$_{C}N$	M_N	26,274	1,897	0.01
	$_{N}M$	20,538	1,497	0.00
N_C	M_N	15,504	506	0.00
	$_{N}M$	17,365	1,340	0.00
Interactions of GvpO with accessory proteins GvpF - GvpM				
$_{N}O$	F_C	56,548	8,401	1.26
	$_{C}F$	13,351	2,008	0.00
O_N	F_C	29,518	2,064	0.18
	$_{C}F$	13,244	1,136	0.00
$_{C}O$	F_N	125,035	7,187	4.00
	$_{N}F$	181,226	19,959	6.24
O_C	F_N	17,692	723	0.00
	$_{N}F$	16,229	654	0.00

transformant		LAU/mm²	σ (LAU/mm²)	Relative fluorecescence (rf)
$_{N}O$	G_C	84,018	6,308	2.36
	$_{C}G$	12,866	420	0.00
O_N	G_C	48,243	5,351	0.87
	$_{C}G$	17,052	2,596	0.00
$_{C}O$	G_N	42,310	2,497	0.64
	$_{N}G$	65,608	9,355	1.54
O_C	G_N	13,769	411	0.00
	$_{N}G$	16,672	851	0.00
$_{N}O$	H_C	75,462	10,197	1.92
	$_{C}H$	16,166	322	0.00
O_N	H_C	45,263	2,354	0.75
	$_{C}H$	15,949	420	0.00
$_{C}O$	H_N	81,350	4,843	2.15
	$_{N}H$	86,017	3,027	2.33
O_C	H_N	18,379	677	0.00
	$_{N}H$	18,969	742	0.00
$_{N}O$	I_C	19,207	1,919	0.00
	$_{C}I$	19,820	1,047	0.00
O_N	I_C	18,845	1,008	0.00
	$_{C}I$	20,944	1,015	0.00
$_{C}O$	I_N	217,184	31,211	6.85
	$_{N}I$	108,712	17,305	2.93
O_C	I_N	26,595	3,932	0.05
	$_{N}I$	21,146	1,424	0.00
$_{N}O$	J_C	58,023	2,375	1.10
	$_{C}J$	22,563	2,695	0.00
O_N	J_C	39,450	1,644	0.43
	$_{C}J$	21,355	1,574	0.00
$_{C}O$	J_N	28,415	1,321	0.04
	$_{N}J$	28,111	2,083	0.04
O_C	J_N	21,395	1,388	0.00
	$_{N}J$	19,947	433	0.00
$_{N}O$	K_C	45,695	4,661	0.63
	$_{C}K$	20,819	767	0.00
O_N	K_C	30,470	570	0.09
	$_{C}K$	21,007	317	0.00
$_{C}O$	K_N	58,649	9,306	1.09
	$_{N}K$	34,302	2,497	0.23
O_C	K_N	19,066	785	0.00
	$_{N}K$	20,901	1,265	0.00
$_{N}O$	L_C	126,105	15,489	3.59
	$_{C}L$	20,524	1,981	0.00
O_N	L_C	55,647	6,188	1.03
	$_{C}L$	19,601	1,130	0.00
$_{C}O$	L_N	280,054	10,160	9.19
	$_{N}L$	43,044	1,625	0.80
O_C	L_N	24,117	1,016	0.02
	$_{N}L$	14,955	444	0.00
$_{N}O$	M_C	45,535	2,869	0.91
	$_{C}M$	14,387	1,300	0.00
O_N	M_C	31,219	2,621	0.31
	$_{C}M$	15,034	1,316	0.00
$_{C}O$	M_N	27,852	1,368	0.17
	$_{N}M$	22,590	946	0.00
O_C	M_N	16,297	239	0.00
	$_{N}M$	15,131	850	0.00
Interaction of GvpF substitutions with GvpA				
A_C	F_E03A_N	254,366	14,575	14.47
	F_E03R_N	229,907	40,082	14.05
	F_E12A_N	248,406	16,983	14.74
	F_E12R_N	290,666	23,606	14.97
	F_E14A_N	265,421	17,761	15.82
	F_E14R_N	241,919	14,398	14.33
	F_D15A_N	219,461	10,943	12.91
	F_D15R_N	205,928	11,495	12.05
	F_E17A_N	274,410	19,959	16,39

transformant		LAU/mm²	σ (LAU/mm²)	Relative fluorecescence (rf)
	F_E17R_N	247,461	19,232	14.68
	F_D19A_N	230,051	6,471	13.58
	F_D19R_N	236,536	11,488	12.00
	F_E21A_N	289,187	11,360	17.33
	F_E21R_N	306,919	13,772	15.86
	F_E27A_N	32,106	2,718	1.10
	F_E27R_N	47,134	2,153	2.08
	F_D45A_N	213,565	27,690	12.98
	F_D45R_N	207,429	43,080	12.58
	F_D48A_N	256,333	52,466	15.78
	F_D48R_N	249,983	44,356	15.36
	F_E50A_N	241,241	48,100	14.79
	F_E50R_N	254,715	43,651	15.67
	F_R51A_N	255,238	62,632	15.09
	F_R51E_N	177,030	3,518	10.16
	F_D53A_N	254,977	12,795	13.69
	F_D53R_N	237,828	17,057	12.70
	F_E54A_N	215,737	11,306	14.30
	F_E54R_N	217,630	10,046	14.43
	F_D55A_N	230,952	16,145	15.38
	F_D55R_N	182,954	14,994	11.97
	F_E57A_N	246,579	12,008	16.48
	F_E57R_N	248,599	27,367	16.63
	F_E65A_N	235,983	19,817	15.73
	F_E65R_N	230,331	10,163	15.33
	F_K68A_N	235,037	5,196	15.67
	F_K68E_N	228,357	10,592	15.19
	F_E70A_N	245,127	14,658	14.51
	F_E70R_N	238,562	8,179	14.09
	F_E71A_N	234,768	17,499	13.85
	F_E71R_N	278,672	12,098	16.63
	F_E72A_N	255,052	6,900	15.14
	F_E72R_N	261,191	14,345	15.52
	F_R73A_N	210,667	6,432	12.33
	F_R73E_N	109,908	5,879	5.95
	F_K85A_N	223,593	7,620	12.91
	F_K85E_N	187,915	43,341	10.85
	F_R88A_N	212,074	26,579	12.37
	F_R88E_N	232,064	59,358	13.63
	F_K91A_N	178,697	29,253	10.27
	F_K91E_N	218,182	42,472	12.76
	F_R95A_N	256,883	10,350	15.20
	F_R95E_N	236,342	39,822	15.35
	F_R98A_N	294,631	15,968	17.76
	F_R98E_N	272,261	21,083	17.83
	F_R102A_N	238,481	7,518	15.49
	F_R102E_N	250,330	17,027	16.31
	F_D123A_N	278,861	22,425	18.29
	F_D123R_N	254,684	32,754	16.61
	F_D124A_N	257,670	11,884	16.82
	F_D124R_N	251,521	12,642	16.40
	F_D154A_N	176,585	10,210	11.21
	F_D154R_N	199,222	5,800	14.04
	F_R155A_N	272,917	20,090	19.60
	F_R155E_N	256,900	53,501	18.40
	F_D184A_N	188,955	8,173	13.27
	F_D184R_N	214,994	11,104	15.23
	F_E185A_N	239,742	11,821	17.10
	F_E185R_N	162,311	9,024	11.25
	F_R213A_N	298,062	17,615	17.13
	F_R213E_N	276,644	14,657	15.83
GvpA dimerization with additional Gvp				
$_N$A	A_C + C	15,073	1,100	0.00
	$_C$A + C	14,327	1,147	0.00
A_N	A_C + C	15,688	1,431	0.00
	$_C$A + C	13,013	1,077	0.00
$_N$A	A_C + N	35,884	7,123	1.28
	$_C$A + N	19,114	9,159	0.40
A_N	A_C + N	32,929	2,001	1.09
	$_C$A + N	25,834	8,000	0.64

transformant		LAU/mm²	σ (LAU/mm²)	Relative fluorecescence (rf)
$_N$A	A_C + O	30,874	2,356	0.96
	$_C$A + O	13,739	3,997	0.06
A_N	A_C + O	27,971	1,451	0.78
	$_C$A + O	16,181	2,367	0.09
$_N$A	A_C + NO	36,627	3,930	1.64
	$_C$A + NO	17,443	10,308	0.50
A_N	A_C + NO	48,505	6,799	2.49
	$_C$A + NO	25,647	1,667	0.85
$_N$A	A_C + CNO	14,637	922	0.00
	$_C$A + CNO	220,807	9,603	5.12
A_N	A_C + CNO	14,870	1,092	0.00
	$_C$A + CNO	13,929	2,429	0.00
